# Immunization with Recombinant Accessory Protein-Deficient SARS-CoV-2 Protects against Lethal Challenge and Viral Transmission

**DOI:** 10.1128/spectrum.00653-23

**Published:** 2023-05-16

**Authors:** Chengjin Ye, Jun-Gyu Park, Kevin Chiem, Piyush Dravid, Anna Allué-Guardia, Andreu Garcia-Vilanova, Paula Pino Tamayo, Vinay Shivanna, Amit Kapoor, Mark R. Walter, James J. Kobie, Richard K. Plemper, Jordi B. Torrelles, Luis Martinez-Sobrido

**Affiliations:** a Disease Intervention and Prevention, and Population Health Programs, Texas Biomedical Research Institute, San Antonio, Texas, USA; b Center for Vaccines and Immunity, The Research Institute at Nationwide Children’s Hospital, Columbus, Ohio, USA; c Department of Microbiology, University of Alabama at Birmingham, Birmingham, Alabama, USA; d Department of Medicine, Division of Infectious Diseases, University of Alabama at Birmingham, Birmingham, Alabama, USA; e Center for Translational Antiviral Research, Institute for Biomedical Sciences, Georgia State University, Atlanta, Georgia, USA; University of Manitoba

**Keywords:** SARS-CoV-2, live-attenuated vaccine, immune protection, viral shedding, viral transmission, coronavirus

## Abstract

Severe acute respiratory syndrome coronavirus 2 (SARS-CoV-2) has led to a worldwide coronavirus disease 2019 (COVID-19) pandemic. Despite the high efficacy of the authorized vaccines, there may be uncertain and unknown side effects or disadvantages associated with current vaccination approaches. Live-attenuated vaccines (LAVs) have been shown to elicit robust and long-term protection by the induction of host innate and adaptive immune responses. In this study, we sought to verify an attenuation strategy by generating 3 double open reading frame (ORF)-deficient recombinant SARS-CoV-2s (rSARS-CoV-2s) simultaneously lacking two accessory ORF proteins (ORF3a/ORF6, ORF3a/ORF7a, and ORF3a/ORF7b). We report that these double ORF-deficient rSARS-CoV-2s have slower replication kinetics and reduced fitness in cultured cells compared with their parental wild-type (WT) counterpart. Importantly, these double ORF-deficient rSARS-CoV-2s showed attenuation in both K18 hACE2 transgenic mice and golden Syrian hamsters. A single intranasal dose vaccination induced high levels of neutralizing antibodies against SARS-CoV-2 and some variants of concern and activated viral component-specific T cell responses. Notably, double ORF-deficient rSARS-CoV-2s were able to protect, as determined by the inhibition of viral replication, shedding, and transmission, against challenge with SARS-CoV-2 in both K18 hACE2 mice and golden Syrian hamsters. Collectively, our results demonstrate the feasibility of implementing the double ORF-deficient strategy to develop safe, immunogenic, and protective LAVs to prevent SARS-CoV-2 infection and associated COVID-19.

**IMPORTANCE** Live-attenuated vaccines (LAVs) are able to induce robust immune responses, including both humoral and cellular immunity, representing a very promising option to provide broad and long-term immunity. To develop LAVs for SARS-CoV-2, we engineered attenuated recombinant SARS-CoV-2 (rSARS-CoV-2) that simultaneously lacks the viral open reading frame 3a (ORF3a) in combination with either ORF6, ORF7a, or ORF7b (Δ3a/Δ6, Δ3a/Δ7a, and Δ3a/Δ7b, respectively) proteins. Among them, the rSARS-CoV-2 Δ3a/Δ7b was completely attenuated and able to provide 100% protection against an otherwise lethal challenge in K18 hACE2 transgenic mice. Moreover, the rSARS-CoV-2 Δ3a/Δ7b conferred protection against viral transmission between golden Syrian hamsters.

## INTRODUCTION

Coronaviruses (CoVs) cause mild to lethal respiratory infections (1, 2). Severe acute respiratory syndrome CoV 2 (SARS-CoV-2) is the etiological agent of the worldwide CoV disease 2019 (COVID-19) pandemic. As of February 2023, more than 672 million cases and over 6.8 million deaths have been confirmed worldwide (https://covid19.who.int/). SARS-CoV-2 belongs to the *Coronaviridae* family, order *Nidovirales*, and is an enveloped positive-sense, single-stranded RNA virus with a large and nonsegmented genome of ~30 kb in length. The SARS-CoV-2 genome encodes a major open reading frame (ORF) frameshifted polyprotein (ORF1a/ORF1ab) and four structural proteins, namely, the spike (S), envelope (E), membrane (M), and nucleocapsid (N) proteins (3). In addition, at least six ORFs encoding accessory proteins (ORF3a, ORF6, ORF7a, ORF7b, ORF8, and ORF10) are interspersed between the structural genes (4). These accessory proteins have been reported to be involved in viral pathogenesis and the regulation of different aspects of the host response to viral infection, including cell apoptosis and pyroptosis, interferon (IFN) signaling, cell cytokine secretion, and immune modulation, among others (5–14).

Subunit-, inactivated-, mRNA-, and vector-based vaccines have been developed for SARS-CoV-2, and the United States Food and Drug Administration (FDA) has authorized the clinical use of several vaccines, including 2 types of mRNA vaccines, 1 adenoviral vector vaccine and 1 adjuvanted S protein nanoparticle vaccine (Novavax COVID-19) (15–21). These vaccines rely on the expression of the viral S protein to induce high levels of neutralizing antibodies against SARS-CoV-2, and neither of them could provide long-term protection. Live-attenuated vaccines (LAVs) have traditionally been highly successful at inducing effective and long-term immunity. Since LAVs most closely resemble a natural infection, and yet have diminished pathogenesis, a comprehensive immune response is induced, calling on B cell and T cell responses to target not only viral glycoproteins but also other viral components. For example, the yellow fever LAV is considered one of the safest and most efficacious vaccines developed to date (22–24). Initial attempts to generate a SARS-CoV-2 LAV using a mutant virus that lacked the furin cleavage site in the S protein showed reduced viral pathogenesis in both K18 hACE2 transgenic mice and golden Syrian hamsters and conferred protection against challenge with the parental virus (25). Another intranasal (i.n.) LAV strategy used a highly attenuated SARS-CoV-2 that incorporated a furin deletion plus an S protein segment with synonymous suboptimal codon pairs (codon-pair deoptimization), which protected hamsters from SARS-CoV-2-associated weight loss (26). Additionally, the codon-pair deoptimization strategy was extended to the entire viral genome, resulting in two recombinant SARS-CoV-2 (rSARS-CoV-2) LAV candidates that induced strong protective immunity in hamsters, blocking all clinical symptoms (27) and eliciting superior mucosal and systemic immunity to SARS-CoV-2 variants (28). However, the attenuated SARS-CoV-2 LAV generated in these studies still have a high risk of reverting, and their efficacies, including protection against infection and transmission, have not been evaluated fully.

Previously, we attempted to develop attenuated rSARS-CoV-2 by deleting single ORF viral accessory proteins (ORF3a, ORF6, ORF7a, ORF7b, and ORF8). However, these single ORF-deficient rSARS-CoV-2s were still lethal in the K18 hACE2 transgenic mouse model, with only the rSARS-CoV-2 ΔORF3a showing attenuation, with a 75% survival rate (29). In this study, we use our previously described reverse genetics system (30) to extend the attenuation strategy by starting with rSARS-CoV-2 ΔORF3a to generate recombinant viruses with a second ORF deletion (ORF3a/ORF6, ORF3a/ORF7a, ORF3a/ORF7b, and ORF3a/ORF8). Finally, the rSARS-CoV-2 harboring deletions of ORF3a/ORF6 (Δ3a/Δ6), ORF3a/ORF7a (Δ3a/Δ7a), or ORF3a/ORF7b (Δ3a/Δ7b) were successfully rescued and demonstrated slower kinetics and reduced fitness in cultured cells. The survival time of K18 hACE2 mice infected with any of the 3 double ORF-deficient rSARS-CoV-2s was significantly increased. However, their survival rate varied from 0% for rSARS-CoV-2 Δ3a/Δ7a, 60% for rSARS-CoV-2 Δ3a/Δ6, to 100% for rSARS-CoV-2 Δ3a/Δ7b. Notably, both humoral and cellular immunity were activated in the surviving K18 hACE2 transgenic mice to protect them against subsequent lethal challenge with parental SARS-CoV-2. Similarly, hamsters vaccinated with the 3 double ORF-deficient rSARS-CoV-2s mounted robust levels of neutralizing antibodies without leading to any body weight changes. Importantly, upon challenge with the parental SARS-CoV-2, all vaccinated hamsters showed decreased viral replication, shedding, and transmission.

Together, our results demonstrate that the double ORF-deficient strategy, particularly, the Δ3a/Δ7b double deletion we have developed in this study, is promising for the development of safe, immunogenic, and protective LAVs for the prophylactic treatment against SARS-CoV-2 infection and associated COVID-19 disease.

## RESULTS

### Generation and *in vitro* characterization of rSARS-CoV-2 with deletions of two accessory proteins.

**FIG 1:**
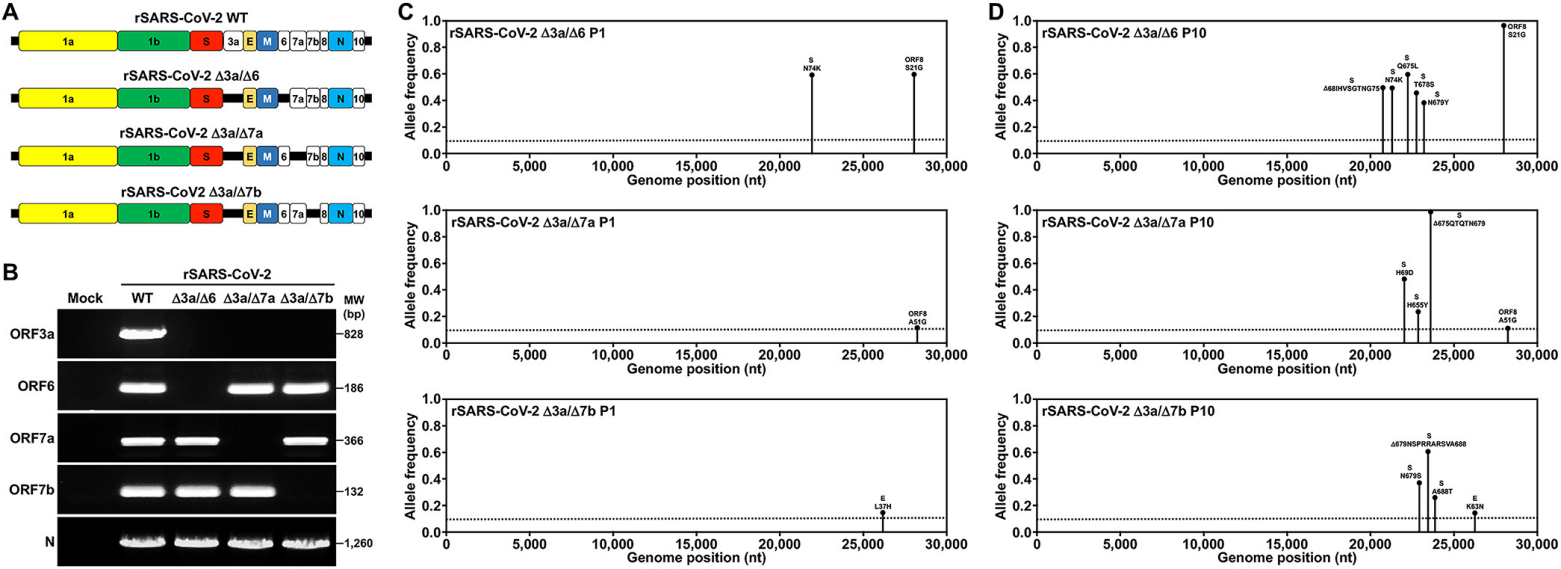
Generation of the double ORF-deficient rSARS-CoV-2 strain. (A) Schematic representations of the double ORF-deficient rSARS-CoV-2 genomes (not drawn to scale). Fragments in the shuttle plasmids that contain a single deletion of the viral ORF6, ORF7a, and ORF7b were released using PpuMI and XhoI restriction enzymes and then were ligated to the PpuMI-XhoI-linearized vector backbone that contains the ORF3a deletion. The fragments in the resultant shuttle plasmids that contain a double deletion of ORF3a/ORF6, ORF3a/ORF7a, and ORF3a/ORF7b were released using BamHI and RsrII digestion and reassembled into the BAC that was linearized with the same restriction enzymes. PCR-positive BAC plasmid colonies were prepared and transfected into Vero E6 cells for virus rescue. (B) Confirmation of the double ORF-deficient rSARS-CoV-2 strain by RT-PCR amplification of ORF3a, ORF6, ORF7a, ORF7b, and N. (C) Deep sequencing analysis of the double ORF-deficient rSARS-CoV-2 P1 genome. Nonreference alleles present in less than 10% of reads are not shown. Amino acid changes respective to rSARS-CoV-2 WT are indicated. (D) Deep sequencing analysis of the double ORF-deficient rSARS-CoV-2 P10 genome. Nonreference alleles present in less than 10% of reads are not shown. Amino acid changes respective to rSARS-CoV-2 WT are indicated.

We attempted four double ORF-deficient rSARS-CoV-2s that simultaneously lacked two accessory proteins (Δ3a/Δ6, Δ3a/Δ7a, Δ3a/Δ7b, and Δ3a/Δ8) using our previously described bacterial artificial chromosome (BAC)-based reverse genetic system (30) (Fig. 1A). Except for the rSARS-CoV-2 Δ3a/Δ8 that did not result in a viable virus, the other three double ORF-deficient rSARS-CoV-2s were rescued and verified by reverse transcriptase PCR (RT-PCR) amplification of the ORF3a, ORF6, ORF7a, ORF7b, and N viral genes (Fig. 1B) and next-generation sequencing (NGS) of the viral genomes. In total, four nonreference alleles were found in these viruses with a frequency greater than 10% in corresponding populations; two nonreference alleles (S N74K and ORF8 S21G) were found in rSARS-CoV-2 Δ3a/Δ6 with a percentage of 58.52% and 57.69%, respectively (Fig. 1C, top); one nonreference allele (ORF8 A51G) was found in rSARS-CoV-2 Δ3a/Δ7a with a percentage of 12.04% (Fig. 1C, middle), and a nonreference allele (E L37H) was found in rSARS-CoV-2 Δ3a/Δ7b with a percentage of 14.94% (Fig. 1C, bottom). After these double ORF-deficient rSARS-CoV-2s were passaged nine times in Vero E6 cells, the P10 viral RNA was subjected to NGS to assess reversions. Only single-amino acid mutations or deletions (68IHVSGTNG75 for rSARS-CoV-2 Δ3a/Δ6, 675QTQTN679 for rSARS-CoV-2 Δ3a/Δ7a, and 679NSPRRARSVA688 for Δ3a/Δ7b) were found in the S glycoprotein of the double ORF-deficient rSARS-CoV-2 (Fig. 1D). However, neither ORF3a nor other respective ORFs reverted.

**FIG 2:**
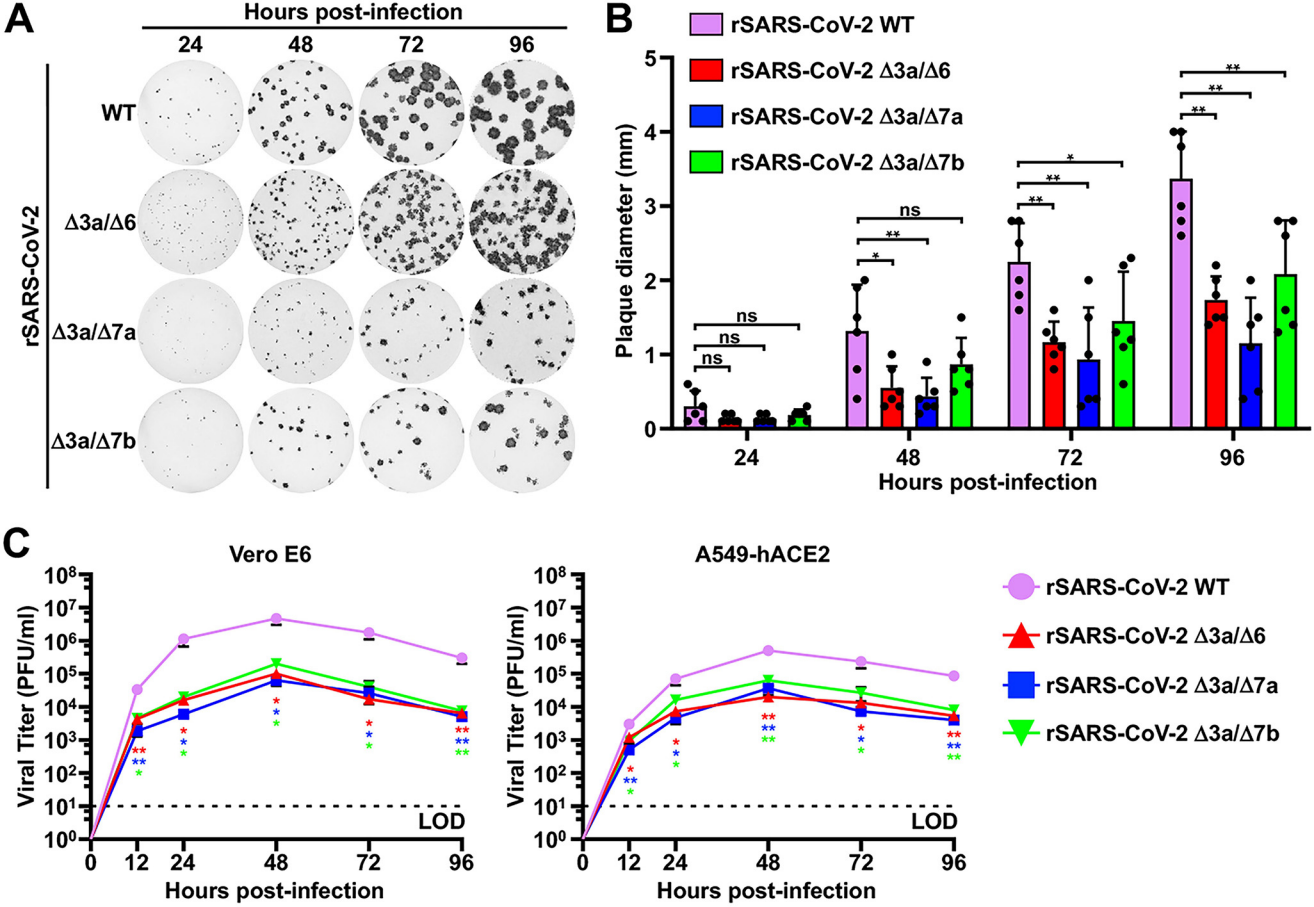
*In vitro* characterization of the double ORF-deficient rSARS-CoV-2 strain. (A) Plaque phenotype. Plaque phenotypes of the WT and double ORF-deficient rSARS-CoV-2 strains in Vero E6 cells. Plaques were visualized by immunostaining with a monoclonal antibody (1C7C7) against the viral N protein. (B) Viral plaque size analysis. Six plaques were randomly selected and measured using a standard ruler (millimeters, mm). Data are presented as mean ± SD, and comparisons of diameter means between indicated groups were performed by one-way ANOVA. *, *P < *0.05; **, *P < *0.01; and ns, not significant. (C) Growth kinetics. Viral growth kinetics of the WT and double ORF-deficient rSARS-CoV-2 strains in Vero E6 (left) and A549-hACE2 (right) cells were measured in triplicate by plaque assay. Dotted lines indicate the limit of detection (LOD). Data are presented as mean ± SEM, and viral titer means of the supernatant collected from the double ORF-deficient rSARS-CoV-2-infected cells are compared with that of the rSARS-CoV-2 WT-infected cells by one-way ANOVA. *, *P < *0.05; and **, *P < *0.01.

Having validated the double ORF-deficient rSARS-CoV-2, we evaluated their plaque morphology in Vero E6 cells at 24, 48, 72, and 96 h postinfection (hpi) (Fig. 2A). All 3 double ORF-deficient rSARS-CoV-2 viral passage 1 (P1) stocks exhibited smaller plaque diameters at 72 and 96 hpi than the rSARS-CoV-2 WT (Fig. 2B). Then, the growth kinetics of these double ORF-deficient rSARS-CoV-2s were evaluated in Vero E6 and A549 expressing human ACE2 (A549 hACE2) cells infected with a multiplicity of infection (MOI) of 0.01 PFU per cell. All double ORF-deficient rSARS-CoV-2 P1 stocks replicated to similar peak titers, which were significantly lower than those reached by rSARS-CoV-2 WT at 24, 48, 72, and 96 hpi in both cell lines (Fig. 2C).

### Characterization of double ORF-deficient rSARS-CoV-2 in K18 hACE2 transgenic mice.

**FIG 3:**
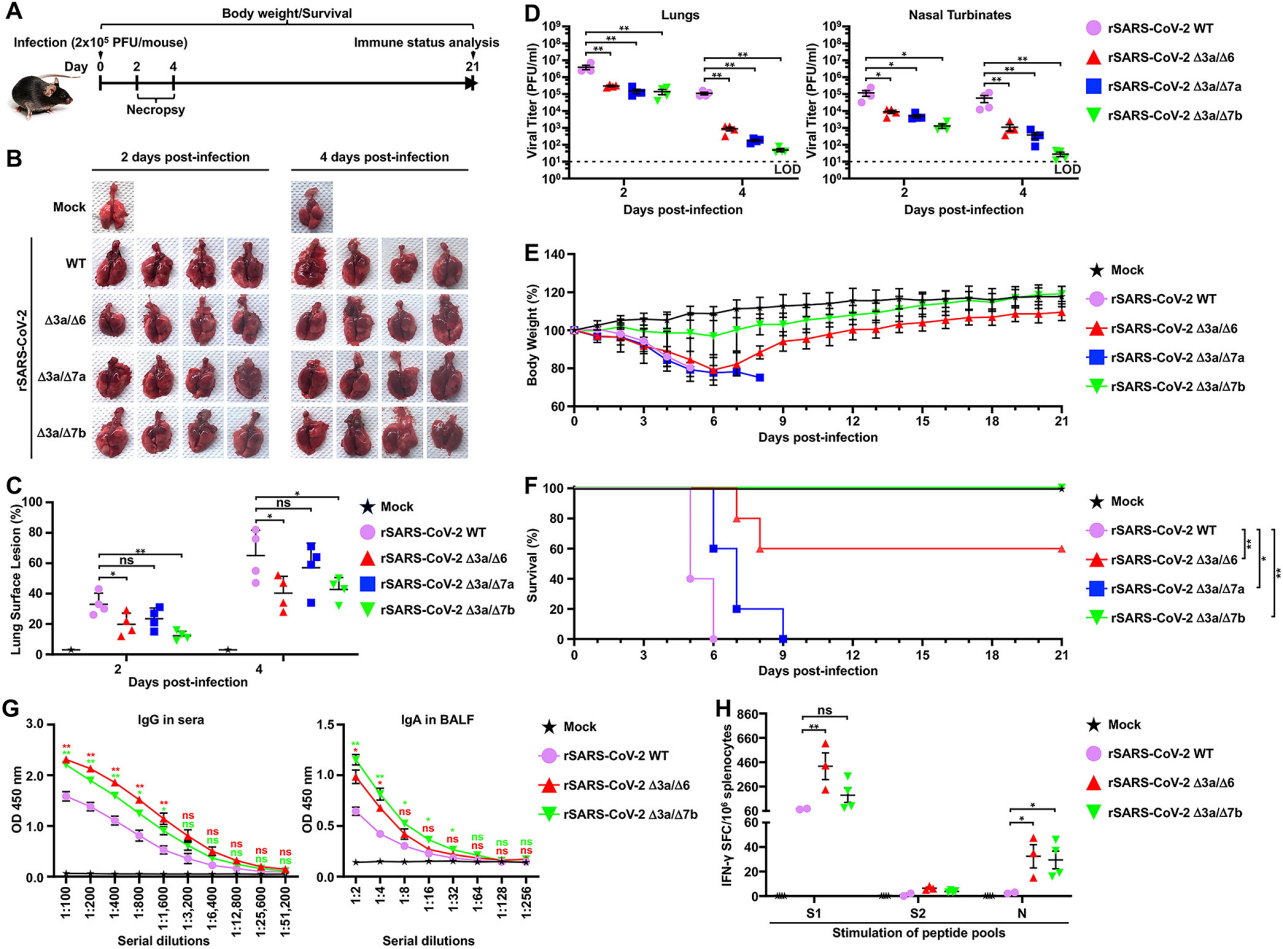
Characterization of double ORF-deficient rSARS-CoV-2 in K18 hACE2 transgenic mice. (A) Schematic representation of the experimental timeline used to infect K18 hACE2 transgenic mice with the WT and double ORF-deficient rSARS-CoV-2 strains. (B) Pathological lesions in the lung surface of K18 hACE2 transgenic mice mock infected or infected (2 × 10^5^ PFU/mouse) with the indicated rSARS-CoV-2 strain at 2 and 4 dpi (*n* = 4/group). (C) Gross pathological lesion scoring on lung images in B using NIH ImageJ. Data are presented as mean ± SD, and comparisons of means between indicated groups are analyzed by one-way ANOVA. *, *P < *0.05; **, *P < *0.01; and ns, not significant. (D) Viral titers in the clarified homogenate of lungs (left) and nasal turbinate (right) of K18 hACE2 transgenic mice infected in B at 2 and 4 dpi. The viral titers in the supernatant of the homogenate were determined in triplicate by plaque assay. Data are presented as mean ± SEM, and comparisons of the means between indicated groups were analyzed by one-way ANOVA. *, *P < *0.05; and **, *P < *0.01. (E) Body weight changes in K18 hACE2 transgenic mice mock infected or infected (2 × 10^5^ PFU/mouse, *n* = 5/group) with the indicated WT or double ORF-deficient rSARS-CoV-2. (F) Survival curves of K18 hACE2 transgenic mice infected in E were calculated and plotted using daily observations for 21 days. The Kaplan-Meier survival analysis with a log rank (Mantel-Cox) test was applied to compare overall survival time. *, *P < *0.05; and **, *P < *0.01. (G) The IgG (sera) and IgA (BALF) against the full-length S glycoprotein in mice that survived in F were tested in triplicate by ELISA at 21 dpi. Sera and BALF collected from the two surviving K18 hACE2 transgenic mice infected with rSARS-CoV-2 WT (10^3^ PFU/mouse, *n* = 5) for 21 days were included as a positive control. Data are presented as mean ± SEM, and means of the double ORF-deficient rSARS-CoV-2 groups are compared with that of the rSARS-CoV-2 WT group by one-way ANOVA. *, *P < *0.05; **, *P < *0.01; and ns, not significant. (H) Splenocytes were isolated from the mice that survived in F at 21 dpi, and IFN-γ-specific spot-forming cells (SFCs) were counted (duplicate) after stimulation with peptide pools of S1, S2, and N using flow cytometry. The splenocytes isolated from the two surviving K18 hACE2 mice infected with rSARS-CoV-2 WT (10^3^ PFU/mouse, *n* = 5) for 21 days were included as a positive control. Data are presented as mean ± SEM, and comparisons of the means between indicated groups are analyzed by one-way ANOVA. *, *P < *0.05; **, *P < *0.01; and ns, not significant.

We next evaluated the replication and pathogenesis of the double ORF-deficient rSARS-CoV-2 in transgenic mice that express human angiotensin converting enzyme 2 (K18 hACE2), which are a common model to use to study SARS-CoV-2 pathogenesis (31, 32). To that end, 5-week-old female K18 hACE2 transgenic mice were infected with the double ORF-deficient rSARS-CoV-2 strain using a dose of 2 × 10^5^ PFU/mouse (Fig. 3A). Mock-infected K18 hACE2 mice and mice infected with the same dose of rSARS-CoV2 WT were used as internal controls. The lungs of infected K18 hACE2 transgenic mice were excised, and gross pathological lesions were analyzed at 2 and 4 days postinfection (dpi) (Fig. 3B). At 2 dpi, the lungs of K18 hACE2 transgenic mice infected with rSARS-CoV-2 WT contained pathological lesions in ~35% of the total lung area, whereas the lungs of K18 hACE2 transgenic mice infected with the double ORF-deficient rSARS-CoV-2 strain had lesions in ≤25% of the total lung area with 19% for rSARS-CoV-2 Δ3a/Δ6, 23% for rSARS-CoV-2 Δ3a/Δ7a, and 12% for rSARS-CoV-2 Δ3a/Δ7b. By 4 dpi, rSARS-CoV-2 WT had induced lesions in ~60% of the total lung area, while double ORF-deficient rSARS-CoV-2 had reduced lesions with ~40% for rSARS-CoV-2 Δ3a/Δ6, ~55% for rSARS-CoV-2 Δ3a/Δ7a, and ~40% for rSARS-CoV-2 Δ3a/Δ7b (Fig. 3C). We also evaluated viral replication of the double ORF-deficient rSARS-CoV-2 strain in the lungs and nasal turbinate of the infected K18 hACE2 transgenic mice. Compared with rSARS-CoV-2 WT, all double ORF-deficient rSARS-CoV-2s replicated to significantly lower titers in the lungs and nasal turbinates of infected mice at both 2 and 4 dpi (Fig. 3D). We further analyzed Th1/Th2/Th17 immune responses and chemokines present in the lungs of K18 hACE2 mice infected with the double ORF-deficient rSARS-CoV-2 (see Fig. S1A in the supplemental material). At 2 dpi, there were no major differences among all virus strains studied, except for the rSARS-CoV-2 Δ3a/Δ7a-infected samples, which showed significantly lower levels of interferon (IFN) responses (IFN-α and IFN-γ). However, by 4 dpi, all the samples from the double ORF-deficient rSARS-CoV-2-infected mice showed a decrease in inducing IFN-α compared with that of rSARS-CoV-2 WT, and the decrease was not observed for IFN-γ. All the double ORF-deficient rSARS-CoV-2 infections also induced significantly lower levels of chemokines by 4 dpi.

Clinical signs demonstrated that K18 hACE2 transgenic mice infected with rSARS-CoV-2 WT started losing body weight at 2 dpi and succumbed to infection by 6 dpi, and a comparable pattern of body weight loss was observed in animals infected with rSARS-CoV-2 Δ3a/Δ6 and rSARS-CoV-2 Δ3a/Δ7a (Fig. 3E). All K18 hACE2 transgenic mice infected with rSARS-CoV-2 Δ3a/Δ7a and 2 out of the 5 mice infected with rSARS-CoV-2 Δ3a/Δ6 succumbed to infection by 9 dpi infection (Fig. 3F). The other three K18 hACE2 transgenic mice infected with rSARS-CoV-2 Δ3a/Δ6 gradually recovered and ultimately survived from viral infection (Fig. 3E and F). Notably, all K18 hACE2 transgenic mice infected with rSARS-CoV-2 Δ3a/Δ7b maintained their initial body weight, with only 2 out of the 5 mice losing a maximum of ~15% of their initial bodyweight before 6 dpi (Fig. 3E). Mortality analysis showed a survival rate of 60% for K18 hACE2 transgenic mice infected with rSARS-CoV-2 Δ3a/Δ6 and 100% survival rate for K18 hACE2 transgenic mice infected with rSARS-CoV-2 Δ3a/Δ7b. All mice infected with rSARS-CoV-2 Δ3a/Δ7a succumbed to viral infection, similar to mice infected with rSARS-CoV-2 WT, although the survival time in mice infected with rSARS-CoV-2 Δ3a/Δ7a was significantly increased compared with mice infected with rSARS-CoV-2 WT (Fig. 3F). At 21 dpi, high levels of immunoglobulin G (IgG) against the full-length viral S protein were detected in the sera of surviving mice, which are higher than that detected in the sera from K18 hACE2 transgenic mice infected with a doubled mouse lethal dose 50 (MLD_50_) of 10^3^ PFU/mouse rSARS-CoV-2 WT (Fig. 3G, left). Furthermore, infection with both rSARS-CoV-2 Δ3a/Δ6 and rSARS-CoV-2 Δ3a/Δ7b induced significantly higher levels of IgA against full-length viral S protein in the bronchoalveolar lavage fluid (BALF) of infected mice than the rSARS-CoV-2 WT (Fig. 3G, right). Importantly, viral Spike 1 (S1), Spike 2 (S2), and N protein-specific IFN-γ secreting cells were detected in the splenocytes of surviving mice (Fig. 3H), and viral S1, E, and M protein-specific cytokine-positive CD4^+^ and CD8^+^ T cells were also identified in both rSARS-CoV-2 Δ3a/Δ6- and rSARS-CoV-2 Δ3a/Δ7b-infected K18 hACE2 transgenic mouse splenocytes (Fig. S1B and S1C).

### Protection of rSARS-CoV-2 Δ3a/Δ7b-vaccinated K18 hACE2 transgenic mice against lethal challenge with SARS-CoV-2.

**FIG 4:**
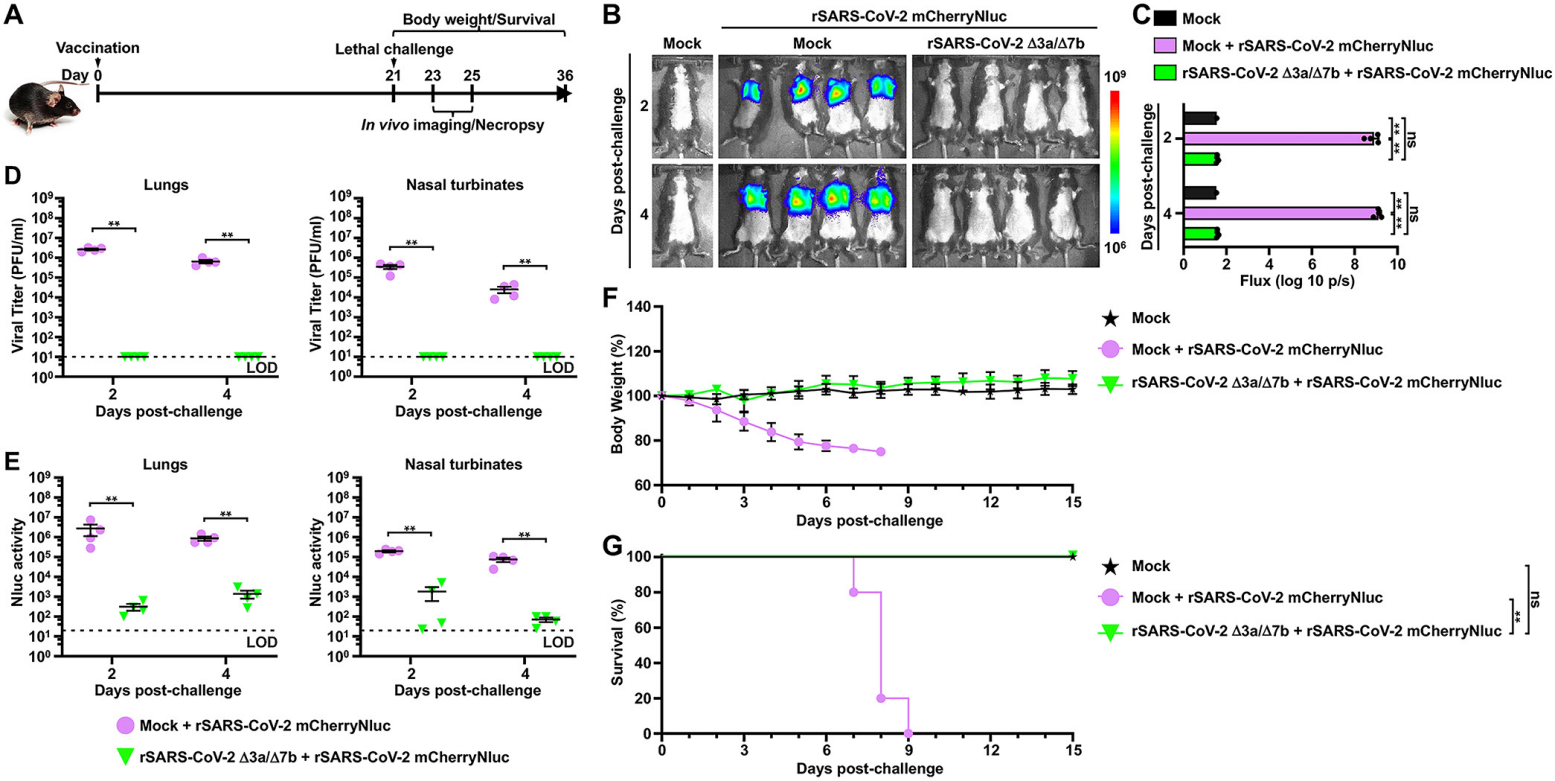
Protection efficacy of rSARS-CoV-2 Δ3a/Δ7b-vaccinated K18 hACE2 transgenic mice against lethal challenge with SARS-CoV-2. (A) Schematic representation of the experimental timeline used for the protection studies with rSARS-CoV-2 Δ3a/Δ7b in K18 hACE2 transgenic mice challenged with rSARS-CoV-2 mCherryNluc. (B) *In vivo* imaging of K18 hACE2 transgenic mice mock vaccinated or vaccinated with rSARS-CoV-2 Δ3a/Δ7b at 2 and 4 days postchallenge with rSARS-CoV-2 mCherryNluc (*n* = 4/group). Mock-vaccinated and mock-challenged K18 hACE2 transgenic mice were used as controls. (C) Quantitative analysis of Nluc expression in K18 hACE2 transgenic mice from B. Data are presented as mean ± SD, and comparisons of the means between indicated groups are analyzed by one-way ANOVA. **, *P < *0.01; and ns, not significant. (D) Viral replication in the lungs and nasal turbinate of K18 hACE2 transgenic mice at 2 and 4 days postchallenge with rSARS-CoV-2 mCherryNluc. The viral titers in the supernatant of tissue homogenates were determined in triplicate by plaque assay. Data are presented as mean ± SEM, and comparisons of the means between indicated groups are analyzed by Student’s *t* test. **, *P < *0.01. (E) Nluc activity in the clarified lung and nasal turbinate homogenates of the K18 hACE2 transgenic mice was determined at 2 and 4 days postchallenge with rSARS-CoV-2 mCherryNluc. Nluc activities in the supernatant of the tissue homogenates were determined in triplicate under a multiplate reader. Data are presented as mean ± SEM, and comparisons of the means between indicated groups are analyzed by Student’s *t* test. **, *P < *0.01. (F) Body weight changes of mock-vaccinated or rSARS-CoV-2 Δ3a/Δ7b-vaccinated K18 hACE2 transgenic mice were monitored for 15 days after challenge with rSARS-CoV-2 mCherryNluc (*n* = 5/group). Mock-vaccinated and mock-challenged K18 hACE2 transgenic mice were used as controls (*n* = 5/group). (G) Survival curves of mock-vaccinated or rSARS-CoV-2 Δ3a/Δ7b-vaccinated K18 hACE2 transgenic mice after challenge with rSARS-CoV-2 mCherryNluc (*n* = 5/group). Mock-vaccinated and mock-challenged K18 hACE2 transgenic mice were used as controls (*n* = 5/group). The Kaplan-Meier survival analysis with a log rank (Mantel-Cox) test was applied to compare overall survival time. **, *P < *0.01; and ns, not significant.

Since infection with rSARS-CoV-2 Δ3a/Δ7b was not lethal in K18 hACE2 transgenic mice (Fig. 3E and F), but it induced robust humoral (Fig. 3G) and cellular (Fig. 3H) immunity, we hypothesized that the K18 hACE2 transgenic mice vaccinated with rSARS-CoV-2 Δ3a/Δ7b would survive a lethal challenge with wild-type virus, confirming the feasibility of rSARS-CoV-2 Δ3a/Δ7b as an LAV strategy. To test this hypothesis, 5-week-old female K18 hACE2 transgenic mice were either mock vaccinated or rSARS-CoV-2 Δ3a/Δ7b vaccinated with 2 × 10^5^ PFU/mouse and challenged intranasally with 10^5^ PFU/mouse of rSARS-CoV-2 mCherryNluc at 21 days postvaccination (Fig. 4A). Viral replication was evaluated using a noninvasive *in vivo* imaging system (IVIS) in the whole organism (Nluc), as described (33). A strong Nluc signal in the lungs of mock-vaccinated mice was detected at 2 and 4 days postchallenge with rSARS-CoV-2 mCherryNluc, whereas no Nluc signal was detected in the lungs of K18 hACE2 transgenic mice vaccinated with rSARS-CoV-2 Δ3a/Δ7b (Fig. 4B and C). In excised lungs, the expression of mCherry was detected readily in mock-vaccinated mice, while no mCherry was expressed in the lungs of mice vaccinated with rSARS-CoV-2 Δ3a/Δ7b (see Fig. S2A and S2B in the supplemental material). Moreover, pathological lesions on the lung surface were much more severe in mock-vaccinated mice than those in rSARS-CoV-2 Δ3a/Δ7b-vaccinated mice (Fig. S2C and S2D). Supernatants of the lung and nasal turbinate homogenates from rSARS-CoV-2 Δ3a/Δ7b-vaccinated mice did not contain infectious virus at both 2 and 4 days postchallenge (Fig. 4D). Significantly decreased Nluc activity was further seen in the supernatants of the lung and nasal turbinate homogenates from rSARS-CoV-2 Δ3a/Δ7b-vaccinated K18 hACE2 transgenic mice at both 2 and 4 days postchallenge compared with that of mock-vaccinate mice (Fig. 4E), which correlated with the *in vivo* imaging data. In the lungs of the mock-vaccinated mice, a significant production of IFN-α and IFN-γ was induced after challenge of rSARS-CoV-2 mCherryNluc (Fig. S2E). In contrast, the IFN responses were not induced in the lungs of rSARS-CoV-2 Δ3a/Δ7b-vaccinated mice at either 2 or 4 days postchallenge, whereas an elevated production of tumor necrosis factor alpha (TNF-α), which is highly related to a protective Th17 response, was induced at 4 days postchallenge (Fig. S2E).

As expected, mock-vaccinated K18 hACE2 transgenic mice lose body weight starting at 2 days postchallenge (Fig. 4F), and all of them succumbed to infection by day 9 postchallenge with rSARS-CoV-2 mCherryNluc (Fig. 4G). In contrast, all mice vaccinated with rSARS-CoV-2 Δ3a/7b showed no clinical signs of disease, including changes in body weight, and all mice (100%) survived the lethal challenge with rSARS-CoV-2 mCherryNluc (Fig. 4F and G).

### Double ORF-deficient rSARS-CoV-2 vaccination prevents viral replication and shedding in hamsters.

**FIG 5:**
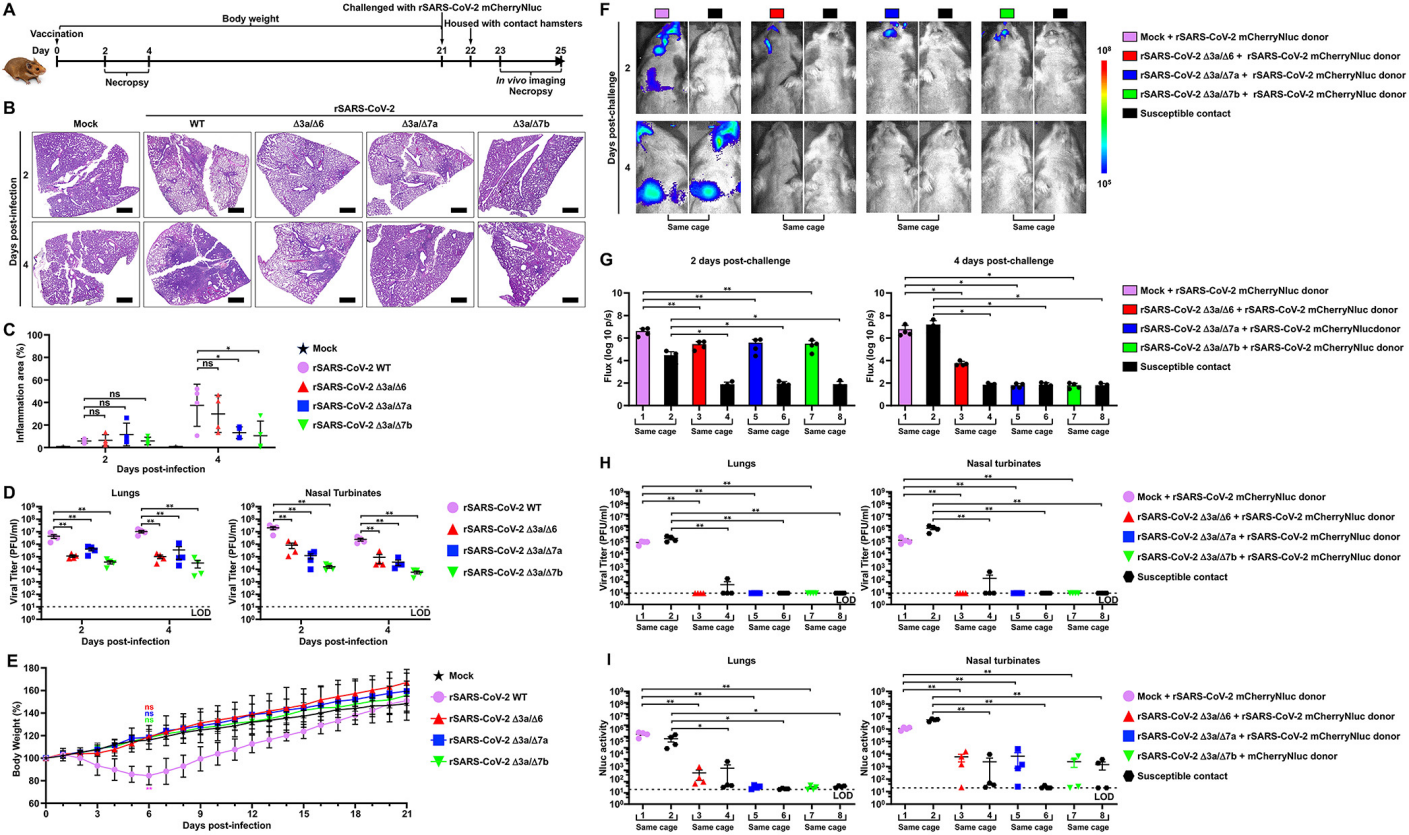
Double ORF-deficient rSARS-CoV-2 vaccination prevents replication and shedding of SARS-CoV-2 in hamsters. (A) Schematic representation of the experimental timeline used for the protection studies with the double ORF-deficient rSARS-CoV-2 strain in hamsters. (B) Representative images of the H&E-stained lungs of the double ORF-deficient rSARS-CoV-2-infected hamsters (*n* = 4/group). Scale bars = 1 mm. (C) Quantitative analysis (% inflammation area) of the extent of bronchointerstitial pneumonia in all of the H&E-stained sections was performed using HALO v3.4 software (*n* = 4/group). Data are presented as mean ± SD, and comparisons of the means between indicated groups are analyzed by one-way ANOVA. *, *P < *0.05; and ns, not significant. (D) Viral titers in the clarified homogenate of lungs (left) and nasal turbinate (right) of double ORF-deficient rSARS-CoV-2-infected hamsters at 2 and 4 dpi (*n* = 4/group). Viral titers in the supernatant of the tissue homogenates were determined in triplicate by plaque assay. Data are presented as mean ± SEM, and comparisons of the means between indicated groups are analyzed by one-way ANOVA. **, *P < *0.01. (E) Body weight changes of mock-infected and WT or double ORF-deficient rSARS-CoV-2-infected hamsters were monitored for 21 days (*n* = 4/group). Data are presented as mean ± SD, and the means of the virus-infected groups were compared with that of the mock control group at 6 dpi by one-way ANOVA. ns, not significant. (F) *In vivo* imaging of the rSARS-CoV-2 mCherryNluc replication in hamsters at 2 and 4 days postchallenge. (G) Quantitative analysis of Nluc expression in hamsters from C at 2 (left) and 4 (right) days postchallenge with rSARS-CoV-2 mCherryNluc by Aura program. Data are presented as mean ± SD, and comparisons of the means between the indicated groups are analyzed by one-way ANOVA. *, *P < *0.05; and **, *P < *0.01. (H) Replication of rSARS-CoV-2 mCherryNluc in the lungs and nasal turbinate of challenged and contact hamsters. Viral titers in the supernatant of the tissue homogenates were determined in triplicate by plaque assay. Data are presented as mean ± SEM, and comparisons of the means between indicated groups are analyzed by one-way ANOVA. **, *P < *0.01. (I) Nluc activity in the clarified lung and nasal turbinate homogenates of infected and contact hamsters. Nluc activity in the supernatant of the tissue homogenates was determined in triplicate under a multiplate reader. Data are presented as mean ± SEM, and comparisons of the means between indicated groups are analyzed by ANOVA. *, *P < *0.05; and **, *P < *0.01.

Next, we sought to investigate the safety and protective efficacy of the double ORF-deficient rSARS-CoV-2 in hamsters, an animal model that resembles the features in humans with mild SARS-CoV-2 infections (34). Five-week-old female golden Syrian hamsters were mock vaccinated or vaccinated (4 × 10^5^ PFU) with the double ORF-deficient rSARS-CoV-2 strain to evaluate viral replication and were monitored daily for body weight (Fig. 5A). The left lung lobes of infected hamsters were collected at 2 and 4 dpi and sectioned to assess inflammation and immunopathology by using hematoxylin and eosin (H&E) staining. The bronchointerstitial pneumonia was focused primarily around bronchioles, terminal airways, and blood vessels, and the severity of pneumonia increased over time (Fig. 5B). However, infection with the double ORF-deficient mutant viruses resulted in a marked reduction in inflammation and reduced severity compared with animals infected with rSARS-CoV-2 WT (Fig. 5C). Furthermore, we also evaluated viral replication of the double ORF-deficient rSARS-CoV-2 strain in the lung and the nasal turbinate of the infected hamsters. Compared with rSARS-CoV-2 WT, all double ORF-deficient rSARS-CoV-2 strains replicated to significantly lower titers in the lungs and nasal turbinate of infected hamsters at both 2 and 4 dpi (Fig. 5D). In addition, infection with rSARS-CoV-2 WT led to an ~15% body weight loss by 6 dpi, and no changes in body weight were observed in hamsters infected with these double ORF-deficient rSARS-CoV-2s, whose body weight were comparable to mock-infected animals at all time points (Fig. 5E).

After 21 days, all vaccinated hamsters were challenged with 2 × 10^5^ PFU of rSARS-CoV-2 mCherryNluc. To assess viral shedding and transmission, each challenge hamster (donor) was housed in the same cage with a susceptible contact hamster at 1 day postchallenge (Fig. 5A). The Nluc signal in all donor and contact hamsters was determined at 2 and 4 days postchallenge. In the donor hamsters, the Nluc signal was detected in the nasal turbinates and lungs of mock-vaccinated hamsters at both time points; to a lesser extent, the Nluc signal was also present in nasal turbinates, but not in the lungs, of the double ORF-deficient rSARS-CoV-2-vaccinated hamsters at 2 days postchallenge, and no Nluc was detected in all of these hamsters at 4 days postchallenge (Fig. 5F and G). In contact hamsters, the Nluc signal was absent in any contact hamsters at 2 and 4 days postchallenge but was readily detectable in all hamsters in contact with mock-vaccinated hamsters at 4 days postchallenge (Fig. 5F and G). After collecting lungs at 4 days postchallenge, we noted strong mCherry expression in the lungs of mock-vaccinated and rSARS-CoV-2 mCherryNluc-infected donor hamsters and their contacts, whereas mCherry fluorescence was significantly decreased in all vaccinated donor hamsters and their respective contacts (see Fig. S3A and S3B in the supplemental material). We next determined virus load in clarified supernatants of lung and nasal turbinate homogenates from both donor and contact hamsters. No detectable infectious virus was present in either tissue in any of the donor hamsters vaccinated with the double ORF-deficient rSARS-CoV-2 strain (Fig. 5H). All contact hamsters were free of virus, except for one contact hamster (~10^2^ PFU/mL) that was cohoused with a hamster vaccinated with rSARS-CoV-2 Δ3a/Δ6 (Fig. 5H). We obtained equivalent results when following Nluc activity in the clarified supernatant of lung and nasal turbinate homogenates (Fig. 5I).

### Double ORF-deficient rSARS-CoV-2 vaccination prevents transmission in hamsters.

**FIG 6:**
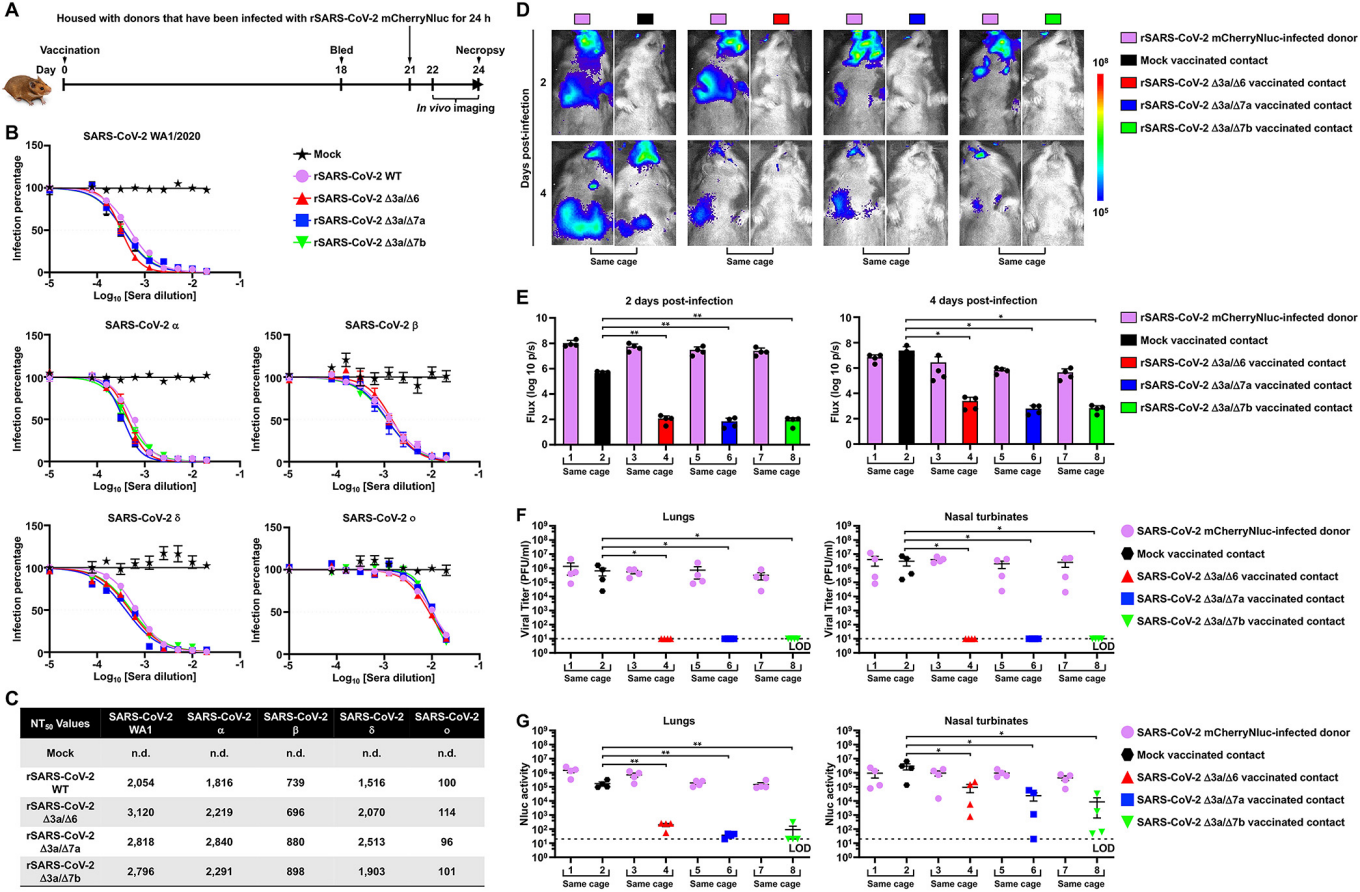
Double ORF-deficient rSARS-CoV-2 vaccination prevents transmission in hamsters. (A) Schematic representation for the experimental timeline used to test the prevention of transmission by double ORF-deficient rSARS-CoV-2 in hamsters. (B) Sera collected at 18 days postvaccination were evaluated for the neutralizing capacity against SARS-CoV-2 WA1/2020, Alpha (α), Beta (β), Delta (δ), and Omicron (ο) VOC by PRMNT assay in quadruplicate (*n* = 4/group). Data are presented as mean ± SEM. (C) Summary of NT_50_ values of sera against the different SARS-CoV-2 VOC. n.d., not determined. (D) *In vivo* imaging of the rSARS-CoV-2 mCherryNluc replication in hamsters at 2 and 4 dpi. (E) Quantitative analysis of Nluc expression in hamsters from D at 2 (left) and 4 (right) dpi by the Aura program. Data are presented as mean ± SD, and comparisons of the means between indicated groups are analyzed by one-way ANOVA. *, *P < *0.05; and **, *P < *0.01. (F) Replication of rSARS-CoV-2 mCherryNluc in the lungs and nasal turbinate of infected donor and vaccinated contact hamsters. Viral titers in the supernatant of the tissue homogenates were determined in triplicate by plaque assay. Data are presented as mean ± SEM, and comparisons of the means between indicated groups are analyzed by One-way ANOVA. *, *P < *0.05. (G) Nluc activity in the clarified lung and nasal turbinate tissue homogenates of infected donor and vaccinated contact hamsters. Nluc activity in the supernatant of the tissue homogenates was determined in triplicate under a multiplate reader. Data are presented as mean ± SEM, and comparisons of the means between indicated groups are analyzed by one-way ANOVA. *, *P < *0.05; and **, *P < *0.01.

Since double ORF-deficient rSARS-CoV-2 vaccination significantly reduced viral replication and shedding after challenge, we sought to explore whether the double ORF-deficient rSARS-CoV-2 strain can provide protection against viral transmission between vaccinated contact hamsters and infected donors. Five-week-old female golden Syrian hamsters were vaccinated with the double ORF-deficient rSARS-CoV-2 strain (4 × 10^5^ PFU), and sera were collected at 18 days postvaccination. Vaccinated contact hamsters were housed with rSARS-CoV-2 mCherryNluc-infected donor hamsters at 21 days postvaccination, and all hamsters were analyzed by *in vivo* imaging and necropsy (Fig. 6A). Sera collected from the double ORF-deficient rSARS-CoV-2-vaccinated hamsters showed a high neutralizing potential against the SARS-CoV-2 WA1 strain and different variants of concern (VOCs) (alpha, α; beta, β; delta, δ; and omicron, ο) (Fig. 6B and C). After rSARS-CoV-2 mCherryNluc-infected (2 × 10^5^ PFU) donor hamsters were cohoused with the double ORF-deficient rSARS-CoV-2-vaccinated contact hamsters, the Nluc signal was readily detected in all donor hamsters at 2 and 4 dpi, whereas no detectable Nluc signal was observed in any of the contact animals at 2 dpi. At 4 dpi, high levels of Nluc signal were present in all mock-vaccinated contact hamsters, but signal was extremely low in all double ORF-deficient rSARS-CoV-2-vaccinated contact hamsters (Fig. 6D and E). In the lungs excised at 4 dpi, mCherry expression was readily detected in all donor hamsters and mock-vaccinated contact hamsters but not in any of double ORF-deficient rSARS-CoV-2-vaccinated contact hamsters (see Fig. S4A and S4B in the supplemental material). When titrating infectious particles in the clarified lung and nasal turbinate homogenates, we found that no infectivity was present in tissues derived from any of the contacts cohoused with the hamsters vaccinated with the double ORF-deficient rSARS-CoV-2 strain (Fig. 6F). Consistent with the viral titer results, Nluc activity in the clarified lung and nasal turbinate homogenates was significantly decreased in all double ORF-deficient rSARS-CoV-2-vaccinated contact hamsters compared with that present seen in mock-vaccinated contacts (Fig. 6G).

## DISCUSSION

LAVs, which can induce broad and long-term host immune responses and activate both innate and adaptive immunity, are attractive for prophylaxes against emerging and re-emerging pathogens that are undergoing rapid antigenic drift, such as SARS-CoV-2 (35). In this study, we used our established BAC-based reverse genetic system (30) to generate 3 potential SARS-CoV-2 LAV candidates by deleting two accessory protein ORFs simultaneously from the viral genome (ORF3a/ORF6, ORF3a/ORF7a, and ORF3a/ORF7b). After being passaged 9 times in Vero E6 cells, the three double ORF-deficient rSARS-CoV-2 strains did not revert, suggesting that the genomes of the double ORF-deficient SARS-CoV-2s are stable *in vitro*. Results also showed that double ORF-deficient rSARS-CoV-2s grow efficiently in Vero E6 cells, which is a cell line approved by the United States FDA for vaccine manufacture and production.

K18 hACE2 transgenic mice were first developed for *in vivo* pathogenicity studies of SARS-CoV (36). Although K18 hACE2 transgenic mice display morbidity and mortality, including efficient replication in the upper and lower respiratory tract and brain, upon SARS-CoV-2 infection (37), it is still unknown how much this K18 hACE2 transgenic mouse model recapitulates a true infection in humans. Since the natural isolate of one of the first SARS-CoV-2 WA1 strains exhibits an MLD_50_ of ~500 PFU (38), we favor the use of the K18 hACE2 transgenic mouse model for testing the safety of live-attenuated rSARS-CoV-2. Using K18 hACE2 transgenic mice, we show that our most attenuated double ORF-deficient virus, rSARS-CoV-2 Δ3a/Δ7b, has an MLD_50_ greater than 2 × 10^5^ PFU, and rSARS-CoV-2 Δ3a/Δ6 showed a MLD_50_ of ~2 × 10^5^ PFU, which is about 400-fold higher than its WT counterpart, suggesting an adequate attenuation of these two rSARS-CoV-2 Δ3a/Δ6 and rSARS-CoV-2 Δ3a/Δ7b LAV candidates. Although all mice infected with rSARS-CoV-2 Δ3a/Δ7b (2 × 10^5^ PFU) succumbed to infection, they still showed a greater survival time than K18 hACE2 transgenic mice infected with rSARS-CoV-2 WT. In addition, we noticed that pathological lesions were observed on the lungs of the 3 double ORF-deficient rSARS-CoV-2-infected K18 hACE2 mice and hamsters at both 2 and 4 dpi, although they were reduced compared with those of the rSARS-CoV-2 WT. However, viral replication of all the double ORF-deficient rSARS-CoV-2s was significantly reduced compared with that of rSARS-CoV-2 WT.

In the case of rSARS-CoV-2 Δ3a/Δ7b-infected K18 hACE2 transgenic mice, 2 of the 5 infected mice had some minimal body weight loss before 6 dpi. Of note, the fully attenuated rSARS-CoV-2 Δ3a/Δ7b strain was able to provide, upon a single intranasal administration, a protective effect in K18 hACE2 transgenic mice challenged with a lethal dose of rSARS-CoV-2 mCherryNluc, and 100% of the rSARS-CoV-2 Δ3a/Δ7b-vaccinated K18 hACE2 transgenic mice survived the challenge with SARS-CoV-2, suggesting that even the most attenuated virus could confer complete protection against a lethal challenge of SARS-CoV-2. The S-specific IgA in the BALF may also contribute to this major protection. However, rSARS-CoV-2 Δ3a/Δ7b-vaccinated mice produced lower levels of IFN responses accompanied with significant decreases in chemokine production and a lower interleukin-6 (IL-6)/IL-10 ratio. These results are consistent with an attenuated cytokine storm, which may explain the reduction in tissue damage observed and the better control of the infection by mice. The later Th1 response observed at 4 dpi indicates that rSARS-CoV-2 Δ3a/Δ7b-vaccinated mice might attenuate the rapid evolution of the detrimental cytokine response observed in the mock-vaccinated mice after challenge of rSARS-CoV-2 mCherryNluc.

Our hamster studies demonstrate that all 3 double ORF-deficient rSARS-CoV-2 strains are highly attenuated and that infection with 4 × 10^5^ PFU results in mild pathological lesions on the lungs without body weight loss, contrary to results with hamsters infected with rSARS-CoV-2 WT, where animals lost up to 15% of their initial body weight by day 6 and had severe pathological lesions, as described previously (30). Furthermore, virus replication in the double ORF-deficient rSARS-CoV-2-vaccinated hamsters after challenge was detected only transiently in the upper respiratory tract (nasal turbinate) at 2 but not at 4 dpi, which is very important for avoiding respiratory complications and pneumonia progression (39). Importantly, viral shedding was impeded in the double ORF-deficient rSARS-CoV-2-vaccinated hamsters as well, as suggested by a lack of viral replication at both 2 and 4 days postchallenge in susceptible contact hamsters. Moreover, vaccination with the double ORF-deficient rSARS-CoV-2s completely abrogated viral transmission from infected donor hamsters, as virus replication was completely undetectable in the vaccinated contact hamsters. The protection could be attributed to the high levels of neutralizing antibody found in sera, including neutralizing antibodies against different VOCs, as well as an activated T cell response. Interestingly, reduced virus replication was seen in infected donor hamsters, which were housed with double ORF-deficient rSARS-CoV-2-vaccinated contact hamsters. We speculate that the close proximity of the vaccinated contact hamster may allow the spread of virus-specific IgA through saliva, feces, and aerosols, which may neutralize virus replication in infected donors. A significant advantage of the double ORF deletion attenuation strategy proposed in our study over other LAVs is that the potential of viral reversion is much lower. Another advantage of our ORF-deficient LAV approach is that other viral S proteins could be expressed instead of the deleted ORF3a and/or ORF7b proteins for the development of bivalent and/or multivalent LAVs, respectively. Together, our data show that the three double ORF-deficient rSARS-CoV-2s, which express all viral structural proteins and yet lack two different accessory protein combinations, are attenuated to different degrees. All double ORF-deficient rSARS-CoV-2 strains induce robust innate and adaptive immune responses, including mucosal immunity, upon a single intranasal administration that protects against subsequent challenge with SARS-CoV-2. Based on the safety profile in K18 hACE2 mice and golden Syrian hamsters, we favor the use of the ORF3a/ORF7b-deficient rSARS-CoV-2 as a LAV for the treatment of SARS-CoV-2 infection. Although the double ORF-deficient rSARS-CoV-2 strain based on the WA1 strain backbone did not induce a comparable level of neutralization antibodies against Omicron compared with other VOCs, it is still reasonable to conclude that the ORF3a/ORF7b-deficient strategy is attractive for the development of SARS-CoV-2 LAVs, as it could be updated easily by substituting the viral S glycoprotein. Alternatively, it will be possible to develop bivalent LAVs by genetically engineering the ORF3a/7b rSARS-CoV-2 to encode additional S proteins from the locus of the viral ORF3a and/or ORF7b proteins. One of the limitations of our current study is the safety profile of the ORF3a/ORF7b-deficient rSARS-CoV-2 strain in immunocompromised hosts. Future experiments in immunocompromised animals are guaranteed to further demonstrate the safety profile of the rSARS-CoV-2 ΔORF3a/7b. Combining attenuation with high immunogenicity and feasibility of update makes the ORF3a/7b-deficient virus an attractive option for its use as safe and protective LAV candidate for prophylaxis of SARS-CoV-2 infection and associated COVID-19 disease. Moreover, the double ORF-deficient SARS-CoV-2 strain represents a valid viral surrogate that could be safely used under less restricted biosafety level 2 (BSL2) laboratories to accelerate research on this important human pathogen, including the identification and characterization of therapeutics or the generation of mutant viruses without gain-of-function safety concerns.

## MATERIALS AND METHODS

### Biosafety.

All *in vitro* and *in vivo* experiments with infectious natural isolates or rSARS-CoV-2s were conducted under appropriate biosafety level 3 (BSL3) and animal BSL3 (ABSL3) laboratories, respectively, at the Texas Biomedical Research Institute. All experiments were approved by the Texas Biomed Institutional Biosafety (IBC) and Animal Care and Use Committees (IACUC).

### Cells, peptides, proteins, antibodies, and viruses.

African green monkey kidney epithelial cells (Vero E6, CRL-1586) were obtained from the American Type Culture Collection (ATCC; Bethesda, MD), and a A549 cell line expressing hACE2 and a Vero E6 cell line expressing hACE2 and TMPRSS2 (Vero AT) were obtained from BEI Resources (NR-53821 and NR-54970). Cells were maintained in Dulbecco’s modified Eagle medium (DMEM) supplemented with 5% (vol/vol) fetal bovine serum (FBS; VWR) and 1% penicillin-streptomycin (Corning). For Vero AT, cells were treated every other passage with 5 μg/mL of puromycin for selection of AT-expressing cells.

A set of 181 peptides spanning the complete S protein of the USA-WA1/2020 strain of SARS-CoV-2 and a set of 59 peptides spanning the complete N protein of USA-WA1/2020 of SARS-CoV-2 were obtained from BEI Resources (NR-52402 and NR-52404, respectively). These peptides are 13 to 20 amino acids long, with 10 overlapping amino acids. The S2 peptide pools contain 93 peptides representing the N-terminal half of the S protein (MFVFLVLLPL to AEHVNNSYE) and the S2 peptide pools contain 88 peptides representing the C-terminal half of the S protein (GAEHVNNSYE to VLKGVKLHYT). Peptides were dissolved in sterile water containing 10% dimethyl sulfoxide (DMSO).

The SARS-CoV-2 S full-length protein was purchased from ACROBiosystems (catalog no. SPN-C52H9), the SARS-CoV-2 E protein was purchased from ThermoFisher Scientific (catalog no. RP-87682), and the SARS-CoV-2 M protein was purchased from ProteoGenix (catalog no. PX-COV-P025).

The following mouse reactive antibodies (clone, catalog no., dilution) from BioLegend, BD Biosciences, and ThermoFisher Scientific were used for the analysis of T cells: CD3-PE/Cyanine7 (145-2C11, 100319, 1:400), IFN-γ-PE/Dazzle 594 (XMG1.2, 505845, 1:400), TNF-α-Brilliant Violet 785 (MP6-XT22, 506341, 1:400), IL-4-Brilliant Violet 711 (11B11, 504133, 1:100), IL-17A Alexa Flour 488 (TC11-18H10, 560221, 1:100), IL-21 Alexa Flour 647 (mhalx21, 51-7213-80, 1:100), CD4-BUV 496 (GK1.5, 612952, 1:400), CD8-BUV737 (53-6.7, 612759, 1:400), IL-10-Brilliant Violet 510 (JES5-16E3, 563277, 1:100), and IL-2-PE (JES6-5H4, 12-7021-82, 1:200). The cross-reactive mouse monoclonal antibody against the SARS-CoV N protein, 1C7C7, was kindly provided by Thomas Moran at the Icahn School of Medicine at Mount Sinai.

The natural isolate SARS-CoV-2 of USA-WA1/2020 (NR-52281) and α (NR-54000), β (NR-54008), δ (NR-55611), and ο (NR-56461) VOCs were obtained from BEI Resources. The rSARS-CoV-2 WT strain was generated previously (30), and the rSARS-CoV-2 expressing mCherryNluc strain (rSARS-CoV-2 mCherryNluc) was generated using the strategy described previously (33).

### Reverse genetics and generation of double ORF-deficient rSARS-CoV-2 strains.

The BAC harboring the entire viral genome of SARS-CoV-2 USA-WA1/2020 strain (accession no. MN985325) was described previously (30). The double deletion of accessory ORF proteins was achieved in viral fragment 1 by inverse PCR using primer pairs containing a BsaI type IIS restriction endonuclease site. All the primer sequences are available upon request. Fragments containing the double deletion of accessory ORF proteins were reassembled into the BAC using BamHI and RsrII restriction endonucleases. Virus rescues were performed as described previously (30, 40). Viral passage 1 (P1) stocks were generated in Vero E6 cells and then further concentrated with polyethylene glycol (System Biosciences; catalog no. LV825A-1) following the manufacturer’s protocol, and then they were aliquoted, titrated, and stored at −80°C.

### Plaque assay and immunostaining.

Confluent monolayers of Vero E6 cells (10^6^ cells/well, 6-well plate format, triplicates) were infected with 10-fold serial diluted viral solutions for 1 h at 37°C. After viral adsorption, cells were overlaid with postinfection media containing 1% low melting agar and were incubated at 37°C. At desired times postinfection, cells were fixed overnight with 10% formaldehyde solution at 4°C. For immunostaining, cells were permeabilized with 0.5% (vol/vol) Triton X-100 in phosphate-buffered saline (PBS) for 15 min at room temperature and immunostained using the N protein 1C7C7 monoclonal antibody (1 μg/mL) and the Vectastain ABC kit (Vector Laboratories), following the manufacturers’ instructions. After immunostaining, plates were photographed under a ChemiDoc system (Bio-Rad). Viral plaque diameters were determined using a ruler as a scale that was photographed together with the 6-well plates.

### Growth kinetics.

Vero E6 or A549-hACE2 cell monolayers (6-well plate, triplicate) were infected with viruses at an MOI of 0.01. After viral absorption for 1 h at 37°C, the viral supernatant was discarded and cells were washed 3 times with PBS. Then, 3 mL of postinfection media (DMEM containing 2% FBS and 1% penicillin-streptomycin-glutamine [PSG]) was added to each well. At the indicated time points (12, 24, 48, 72, and 96h) postinfection, the supernatant (500 μL) was collected and stored at −80°C. All supernatants were finally subjected to viral titration by plaque assay.

### RNA extraction and RT-PCR.

Total RNA from mock- or virus-infected (MOI of 0.01) Vero E6 cells (10^6^ cells/well, 6-well plate format) was extracted with TRIzol reagent (Thermo Fisher Scientific) according to the manufacturer’s instructions. RT-PCR amplification of viral ORF3a, ORF6, ORF7a, ORF7b, ORF8 and N genes was performed using Super Script II reverse transcriptase (Thermo Fisher Scientific) and an expanded high-fidelity PCR system (Sigma-Aldrich). Amplified DNA products were subjected to 1.0% agarose gel analysis. All primer sequences used for RT-PCR are available upon request.

### Deep sequencing.

Sequencing libraries were generated using the Kapa RNA HyperPrep kit with a 45-min adapter ligation incubation, including 6 cycles of PCR with 100 ng viral RNA and 7 mM adapter. Samples were sequenced with an Illumina HiSeq X instrument. Raw reads were quality filtered using Trimmomatic v0.39 (41) and mapped to a SARS-CoV-2 USA-WA1/2020 strain reference genome (GenBank accession no. MN985325) with Bowtie 2 v2.4.1 (42). Genome coverage was quantified with Mosdepth v0.2.6 (43). We genotyped each sample for low-frequency variants with LoFreq* v2.1.3.1 (44) and filtered sites with less than 100× read depth or minor allele frequencies less than 1%. Finally, we used SnpEff v4.3t (45) to identify the impact of potential variants on the protein-coding regions in the SARS-CoV-2 reference genome.

### Virus passages *in vitro*.

Confluent monolayers of Vero E6 cells (10^6^ cells/well, 6-well plate format, triplicates) were infected with rSARS-CoV-2 WT, Δ3a/Δ6, Δ3a/Δ7a, and Δ3a/Δ7b P1 stocks at an MOI of 0.01. At 72 hpi, 5 μL of the supernatants was collected and used to infect another set of confluent Vero E6 cells (10^6^ cells/well, 6-well plate format, triplicates). After the passage procedure was repeated 9 times, the resultant supernatants were labeled as P10. Then, both P1 and P10 stocks were characterized by plaque assay and immunostaining.

### Animal experiments.

All animal protocols were approved by Texas Biomedical Research Institute IACUC. Five-week-old female K18 hACE2 transgenic mice were purchased from The Jackson Laboratory, and 5-week-old female golden Syrian hamsters were purchased from Charles River Laboratories. All the animals were maintained in the animal facility at Texas Biomedical Research Institute under specific-pathogen-free conditions. Animal infection was performed by intranasal inoculation after animals were anesthetized following gaseous sedation in an isoflurane chamber.

### Pathogenicity analysis of the double ORF-deficient rSARS-CoV-2 strain in K18 hACE2 transgenic mice.

Five-week-old K18 hACE2 transgenic mice (*n* = 8) were infected intranasally (i.n.) (2 × 10^5^ PFU) with the double ORF-deficient rSARS-CoV-2 or rSARS-CoV-2 WT strain. At 2 and 4 dpi, four mice per group were sacrificed humanely to collect lungs and nasal turbinates, and gross images of lungs were taken using an iPhone 6s (Apple). Then, nasal turbinates and lungs were homogenized and processed as described previously (31). Tissue homogenates were centrifuged at 12,000 × *g* for 5 min at 4°C, and the clarified supernatants were collected for further measurement of viral titers and cytokine and chemokine induction. Another set of 5-week-old K18 hACE2 transgenic mice (*n* = 5) were mock infected or infected (i.n.; 2 × 10^5^ PFU) with the double ORF-deficient rSARS-CoV-2 or rSARS-CoV-2 WT strain to evaluate body weight changes and survival rate daily for 21 days. The surviving mice were bled to collect serum to assess total IgG levels against the viral full-length S protein. Afterward, mice were sacrificed to collect spleens for an analysis of T cell responses in splenocytes.

### Immunization with rSARS-CoV-2 Δ3a/Δ7b protected K18 hACE2 transgenic mice from lethal challenge of rSARS-CoV-2 mCherryNluc.

Five-week-old K18 hACE2 transgenic mice (*n* = 8) were mock vaccinated or vaccinated with rSARS-CoV-2 Δ3a/Δ7b (i.n.; 2 × 10^5^ PFU). At 21 days postvaccination, all vaccinated mice were challenged i.n. with rSARS-CoV-2 mCherryNluc (10^5^ PFU). At 2 and 4 days postchallenge, mice (*n* = 4) were anesthetized with isoflurane and imaged immediately under an *in vivo* imaging system (IVIS; AMI HTX) after being retro-orbitally injected with 100 μL of Nano-Glo luciferase substrate (Promega). The bioluminescence data acquisition and analysis were performed using the Aura program (Spectral Imaging Systems). Flux measurements were acquired from the region of interest. Then, lungs were excised to analyze mCherry expression under the IVIS, and brightfield images were taken using an iPhone 6s for pathological lesion analysis. Finally, nasal turbinates and lungs were homogenized as described above and the clarified supernatants of homogenate were collected for further measurement of viral titers, Nluc activity, and cytokine and chemokine induction. Another set of 5-week-old K18 hACE2 transgenic mice (*n* = 5) were mock vaccinated or vaccinated (i.n.; 2 × 10^5^ PFU) with rSARS-CoV-2 Δ3a/Δ7b and challenged (i.n.; 10^5^ PFU) with rSARS-CoV-2 mCherryNluc at 21 days postvaccination. Body weight and survival rate were assessed by daily monitoring for 15 days postchallenge.

### Histopathology analysis of the double ORF-deficient rSARS-CoV-2 strain in hamsters.

Five-week-old golden Syrian hamsters (*n* = 4) were mock infected or infected (i.n.; 4 × 10^5^ PFU) with the double ORF-deficient rSARS-CoV-2 or rSARS-CoV-2 WT strain. At 2 and 4 dpi, four hamsters per group were sacrificed humanely to collect lungs and nasal turbinate. Left lung lobes were fixed in 10% formalin, processed, and stained with hematoxylin and eosin (H&E) to determine the lung inflammation score (% inflammation area involved) and pulmonary pathology. The percentage of bronchointerstitial pneumonia in all of the H&E-stained sections was quantified, in a blind manner, by a veterinary pathologist using HALO v3.4 software. Nasal turbinate and rest of the lung lobes were homogenized and processed as described (31). Tissue homogenates were centrifuged at 12,000 × *g* for 5 min at 4°C, and clarified supernatants were collected for further measurement of viral titers.

### Immunization of hamsters with the double ORF-deficient rSARS-CoV-2 strain prevented viral replication and shedding.

Five-week-old golden Syrian hamsters (*n* = 4) were mock vaccinated or vaccinated (i.n.; 4 × 10^5^ PFU) with the double ORF-deficient rSARS-CoV-2 strain. Mock-vaccinated and vaccinated hamsters were challenged (i.n.; 2 × 10^5^ PFU) with rSARS-CoV-2 mCherryNluc at 21 days postvaccination and then housed with nontreated susceptible contact hamsters (*n* = 4). At 2 and 4 days postchallenge, all hamsters were anesthetized and imaged immediately after intraperitoneal injection of 200 μL of Nano-Glo luciferase substrate. At 4 days postchallenge, all hamsters were euthanized and their lungs were excised. Reporter mCherry expression in the lungs was evaluated by IVIS as described previously (46). Thereafter, all lungs and nasal turbinates were collected and homogenized in 2 mL of PBS, and viral titers and Nluc activity were determined in the clarified supernatants of the lung and nasal turbinate homogenates (46).

### Immunization of hamsters with the double ORF-deficient rSARS-CoV-2 strain prevented viral transmission.

Five-week-old golden Syrian hamsters (*n* = 4) were mock vaccinated or vaccinated (i.n.; 4 × 10^5^ PFU) with the double ORF-deficient rSARS-CoV-2 strain. At 18 days postvaccination, all hamsters were bled and sera were isolated for evaluation of neutralization capacity by plaque reduction microneutralization (PRMNT) assay, as described previously (47). At 21 days postvaccination, mock-vaccinated and vaccinated hamsters were housed with donor hamsters, which had been infected (i.n.; 2 × 10^5^ PFU) with rSARS-CoV-2 mCherryNluc for 24 h. At 2 and 4 dpi with rSARS-CoV-2 mCherryNluc, all hamsters were anesthetized and imaged immediately after intraperitoneal injection of 200 μL of Nano-Glo luciferase substrate. At 4 dpi with rSARS-CoV-2 mCherryNluc, all hamsters were euthanized and their lungs were excised. Reporter mCherry expression in the lungs of hamsters were evaluated under an IVIS, as previously described above. Thereafter, all lungs and nasal turbinates were collected and homogenized in 2 mL of PBS, and viral titers and Nluc activity were determined in the clarified supernatant of lung and nasal turbinate homogenate, as previously described above.

### Enzyme linked immunospot (ELISPOT) assay.

Spleens of K18 hACE2 transgenic mice were collected aseptically at 21 dpi and minced by pressing through cell strainers. Red blood cells were removed by incubation in 0.84% ammonium chloride, and following a series of washes in RPMI 1640, cells were resuspended in RPMI 1640 supplemented with 2 mM l-glutamine, 1 mM sodium pyruvate, 10 mM HEPES, 100 U/mL penicillin, 100 μg/mL streptomycin, and 10% fetal bovine serum. Antigen-specific T cells secreting IFN-γ were enumerated using the anti-mouse IFN-γ ELISPOT assay (U-Cytech; catalog no. CT317-PB5). Cells were plated in 96-well polyvinylidene difluoride (PVDF) plates at 2 × 10^5^ per well in duplicate and stimulated separately with SARS-CoV-2 peptide pools (2 μg/mL), Concanavalin-A (5 μg/mL; Sigma) as a positive control, or media alone as a negative control. The plates were incubated for 42 to 48 h and then developed according to the manufacturer’s instructions. The number of spot-forming cells (SFCs) were measured using an automatic counter (Immunospot).

### Flow cytometric analysis of intracellular cytokine production.

For the detection of SARS-CoV-2-specific intracellular cytokine production, 10^6^ splenocytes were stimulated in 96-well round-bottom plates with an S1 peptide pool (5 μg/mL); purified E (10 μg/mL) or M (1.5 μg/mL) proteins; or media alone or phorbol myristate acetate (PMA)-ionomycin (BioLegend) as negative and positive controls, respectively, for 5 h in the presence of GolgiPlug (BD Biosciences). Following incubation, cells were surface stained for CD3, CD4, and CD8 for 30 min at 4°C, fixed and permeabilized using the cytofix/cytoperm kit (BD Biosciences), and intracellularly stained for IFN-γ, TNF-α, IL-2, IL-17A, IL-21, IL-10, and IL-4 for 30 min at room temperature. Dead cells were identified and removed using the Live/Dead fixable near-infrared (IR) dead cell stain kit (Invitrogen). A positive response was defined as >3 times the background of the negative-control sample. The percentage of cytokine-positive cells was then calculated by subtracting the frequency of positive events in the negative-control samples from that of the test samples. Events were collected on a BD LSRFortessa X-20 flow cytometer following compensation with UltraComp eBeads (Invitrogen). Data were analyzed using FlowJo v10 (Tree Star).

### Plaque reduction microneutralization (PRMNT) assay.

All serum samples were diluted serially by 2-fold (starting dilution of 1:50) and mixed with an equal volume of DMEM containing approximately 200 PFU/well SARS-CoV-2 USA-WA1/2020 or VOC of α, β, δ, and ο (quadruplicate) in a 96-well plate. After incubation at 37°C for 1 h with constant rotation, mixtures were transferred to confluent monolayers (4 × 10^5^ cells/well, quadruplicates) of Vero E6 cells (Vero AT cells for ο), as described previously (47). The mixtures were replaced by fresh DMEM containing 2% FBS after incubation at 37°C for another 1 h. At 24 hpi, cells were fixed in 10% formalin solution overnight and immunostained with a SARS-CoV cross-reactive N protein monoclonal antibody (1C7C7) to visualize the plaques, as described previously (47). The number of plaques were measured by using an ELISPOT assay, and viral neutralization was quantified using a sigmoidal dose-response curve. Mock-infected cells and SARS-CoV-2-infected cells in the absence of serum were included as internal controls. Neutralizing titer at 50% inhibition (NT_50_) was calculated for each serum sample.

### Evaluation of lung pathological lesions.

Macroscopic pathology scoring was evaluated using ImageJ software to determine the percentage of the total surface area of the mouse lungs (dorsal and ventral view) affected by consolidation, congestion, and atelectasis, as described previously (48). Left lung lobes from hamsters were fixed in 10% formalin, processed, and stained with H&E to examine inflammation score and pulmonary pathology. The extent of bronchointerstitial pneumonia across the groups was quantified using HALO software v3.4 (Indica Labs).

### Multiplex cytokine and chemokine assay.

Multiple cytokines and chemokines (IFN-α, IFN-γ, IL-6, IL-10, IL-17A, monocyte chemoattractant protein 1 [MCP-1], RANTES, and TNF-α) were measured using a custom 8-plex panel mouse ProcartaPlex assay (ThermoFisher Scientific; catalog no. PPX-08-MXGZGFX), following the manufacturer’s instructions. The assay was performed in a BSL3 laboratory, and samples were decontaminated by an overnight incubation in 10% formaldehyde solution before readout on a Luminex 100/200 system running on Xponent v4.2 with the following parameters: gate 5,000 to 25,000, 50 μL of sample volume, 50 events per bead, sample timeout 120 s, and low photomultiplier tube (PMT) (LMX100/200: Default). Acquired data were analyzed using ProcartaPlex analysis software v1.0.

### Enzyme-linked immunosorbent assay (ELISA).

ELISA plates were coated with 100 ng/well of the viral S protein in 50 μL of PBS overnight at 4°C. After being blocked by 2.5% BSA for 1 h at 25°C, the plates were washed 3 times with PBS containing 0.1% Tween 20 (PBST) and then incubated (triplicate) with 2-fold serial diluted samples at 25°C (starting dilution of 1:50 for serum and starting dilution of 1:2 for BALF). After 2 h of incubation, plates were washed 3 times with PBST and then incubated with a horseradish peroxidase (HRP)-conjugated goat anti-mouse secondary antibody (ThermoFisher Scientific; catalog no. 31430) at 25°C. After 1 h, plates were washed 3 times and then developed by adding 100 μL/well of 3,3′,5,5′-tetramethylbenzidine (TMB)-ELISA substrate (ThermoFisher Scientific; catalog no. 34029). Reactions were stopped after 10 min by adding 50 μL/well of 3 M H_2_SO_4_ to all the wells. ELISA plates were evaluated with a plate reader (Bio-Tek) at an absorbance of 450 nm.

### Statistical analysis.

A two-tailed Student’s *t* test was used to compare the mean between two groups, one-way analysis of variance (ANOVA) with *post hoc* Dunnett’s multiple-comparison test (versus control) was executed for the mean comparisons between multiple groups and across time, and the Kaplan-Meier survival analysis with a log rank (Mantel-Cox) test was applied to compare overall survival time (GraphPad Prism v8.0). *P* values less than 0.05 (*P < *0.05) were considered statistically significant (*, *P < *0.05; **, *P < *0.01; ns, *P > *0.05).

### Data availability.

All the recombinant viruses and BAC-based reverse genetics described in the study are available online at the following website: https://www.txbiomed.org/services-2/virus-request/.

## References

[1] Moriyama M, Hugentobler WJ, Iwasaki A. 2020. Seasonality of respiratory viral infections. Annu Rev Virol 7:83–101. doi:10.1146/annurev-virology-012420-022445. 32196426

[2] Cui J, Li F, Shi ZL. 2019. Origin and evolution of pathogenic coronaviruses. Nat Rev Microbiol 17:181–192. doi:10.1038/s41579-018-0118-9. 30531947 PMC7097006

[3] Yao H, Song Y, Chen Y, Wu N, Xu J, Sun C, Zhang J, Weng T, Zhang Z, Wu Z, Cheng L, Shi D, Lu X, Lei J, Crispin M, Shi Y, Li L, Li S. 2020. Molecular architecture of the SARS-CoV-2 virus. Cell 183:730–738.e13. doi:10.1016/j.cell.2020.09.018. 32979942 PMC7474903

[4] Hu B, Guo H, Zhou P, Shi ZL. 2021. Characteristics of SARS-CoV-2 and COVID-19. Nat Rev Microbiol 19:141–154. doi:10.1038/s41579-020-00459-7. 33024307 PMC7537588

[5] Ren Y, Shu T, Wu D, Mu J, Wang C, Huang M, Han Y, Zhang X-Y, Zhou W, Qiu Y, Zhou X. 2020. The ORF3a protein of SARS-CoV-2 induces apoptosis in cells. Cell Mol Immunol 17:881–883. doi:10.1038/s41423-020-0485-9. 32555321 PMC7301057

[6] Miorin L, Kehrer T, Sanchez-Aparicio MT, Zhang K, Cohen P, Patel RS, Cupic A, Makio T, Mei M, Moreno E, Danziger O, White KM, Rathnasinghe R, Uccellini M, Gao S, Aydillo T, Mena I, Yin X, Martin-Sancho L, Krogan NJ, Chanda SK, Schotsaert M, Wozniak RW, Ren Y, Rosenberg BR, Fontoura BMA, García-Sastre A. 2020. SARS-CoV-2 Orf6 hijacks Nup98 to block STAT nuclear import and antagonize interferon signaling. Proc Natl Acad Sci USA 117:28344–28354. doi:10.1073/pnas.2016650117. 33097660 PMC7668094

[7] Zhou Z, Huang C, Zhou Z, Huang Z, Su L, Kang S, Chen X, Chen Q, He S, Rong X, Xiao F, Chen J, Chen S. 2021. Structural insight reveals SARS-CoV-2 ORF7a as an immunomodulating factor for human CD14(+) monocytes. iScience 24:102187. doi:10.1016/j.isci.2021.102187. 33615195 PMC7879101

[8] Shemesh M, Aktepe TE, Deerain JM, McAuley JL, Audsley MD, David CT, Purcell DFJ, Urin V, Hartmann R, Moseley GW, Mackenzie JM, Schreiber G, Harari D. 2021. SARS-CoV-2 suppresses IFNbeta production mediated by NSP1, 5, 6, 15, ORF6 and ORF7b but does not suppress the effects of added interferon. PLoS Pathog 17:e1009800. doi:10.1371/journal.ppat.1009800. 34437657 PMC8389490

[9] Park MD. 2020. Immune evasion via SARS-CoV-2 ORF8 protein? Nat Rev Immunol 20:408. doi:10.1038/s41577-020-0360-z. PMC7273379 32504060

[10] Wang L, Liu C, Yang B, Zhang H, Jiao J, Zhang R, Liu S, Xiao S, Chen Y, Liu B, Ma Y, Duan X, Guo Y, Guo M, Wu B, Wang X, Huang X, Yang H, Gui Y, Fang M, Zhang L, Duo S, Guo X, Li W. 2022. SARS-CoV-2 ORF10 impairs cilia by enhancing CUL2ZYG11B activity. J Cell Biol 221:e202108015. doi:10.1083/jcb.202108015. 35674692 PMC9184850

[11] Han L, Zheng Y, Deng J, Nan M-L, Xiao Y, Zhuang M-W, Zhang J, Wang W, Gao C, Wang P-H. 2022. SARS-CoV-2 ORF10 antagonizes STING-dependent interferon activation and autophagy. J Med Virol 94:5174–5188. doi:10.1002/jmv.27965. 35765167 PMC9350412

[12] Hachim A, Gu H, Kavian O, Mori M, Kwan MYW, Chan WH, Yau YS, Chiu SS, Tsang OTY, Hui DSC, Mok CKP, Ma FNL, Lau EHY, Amarasinghe GK, Qavi AJ, Cheng SMS, Poon LLM, Peiris JSM, Valkenburg SA, Kavian N. 2022. SARS-CoV-2 accessory proteins reveal distinct serological signatures in children. Nat Commun 13:2951. doi:10.1038/s41467-022-30699-5. 35618731 PMC9135746

[13] Arshad N, Laurent-Rolle M, Ahmed WS, Hsu JC-C, Mitchell SM, Pawlak J, Sengupta D, Biswas KH, Cresswell P. 2023. SARS-CoV-2 accessory proteins ORF7a and ORF3a use distinct mechanisms to down-regulate MHC-I surface expression. Proc Natl Acad Sci USA 120:e2208525120. doi:10.1073/pnas.2208525120. PMC9910621 36574644

[14] Wong LR, Perlman S. 2022. Immune dysregulation and immunopathology induced by SARS-CoV-2 and related coronaviruses—are we our own worst enemy? Nat Rev Immunol 22:47–56. doi:10.1038/s41577-021-00656-2. 34837062 PMC8617551

[15] Kumar S, Basu M, Ghosh P, Ansari A, Ghosh MK. 2023. COVID-19: clinical status of vaccine development to date. Br J Clin Pharmacol 89:114–149. doi:10.1111/bcp.15552. 36184710 PMC9538545

[16] Mabrouk MT, Huang WC, Martinez-Sobrido L, Lovell JF. 2022. Advanced materials for SARS-CoV-2 vaccines. Adv Mater 34:e2107781. doi:10.1002/adma.202107781. 34894000 PMC8957524

[17] Jackson LA, Anderson EJ, Rouphael NG, Roberts PC, Makhene M, Coler RN, McCullough MP, Chappell JD, Denison MR, Stevens LJ, Pruijssers AJ, McDermott A, Flach B, Doria-Rose NA, Corbett KS, Morabito KM, O'Dell S, Schmidt SD, Swanson PA, Padilla M, Mascola JR, Neuzil KM, Bennett H, Sun W, Peters E, Makowski M, Albert J, Cross K, Buchanan W, Pikaart-Tautges R, Ledgerwood JE, Graham BS, Beigel JH, mRNA-1273 Study Group. 2020. An mRNA vaccine against SARS-CoV-2—preliminary report. N Engl J Med 383:1920–1931. doi:10.1056/NEJMoa2022483. 32663912 PMC7377258

[18] Mendonca SA, Lorincz R, Boucher P, Curiel DT. 2021. Adenoviral vector vaccine platforms in the SARS-CoV-2 pandemic. NPJ Vaccines 6:97. doi:10.1038/s41541-021-00356-x. 34354082 PMC8342436

[19] Keech C, Albert G, Cho I, Robertson A, Reed P, Neal S, Plested JS, Zhu M, Cloney-Clark S, Zhou H, Smith G, Patel N, Frieman MB, Haupt RE, Logue J, McGrath M, Weston S, Piedra PA, Desai C, Callahan K, Lewis M, Price-Abbott P, Formica N, Shinde V, Fries L, Lickliter JD, Griffin P, Wilkinson B, Glenn GM. 2020. Phase 1–2 trial of a SARS-CoV-2 recombinant spike protein nanoparticle vaccine. N Engl J Med 383:2320–2332. doi:10.1056/NEJMoa2026920. 32877576 PMC7494251

[20] Corbett KS, Edwards DK, Leist SR, Abiona OM, Boyoglu-Barnum S, Gillespie RA, Himansu S, Schäfer A, Ziwawo CT, DiPiazza AT, Dinnon KH, Elbashir SM, Shaw CA, Woods A, Fritch EJ, Martinez DR, Bock KW, Minai M, Nagata BM, Hutchinson GB, Wu K, Henry C, Bahl K, Garcia-Dominguez D, Ma L, Renzi I, Kong W-P, Schmidt SD, Wang L, Zhang Y, Phung E, Chang LA, Loomis RJ, Altaras NE, Narayanan E, Metkar M, Presnyak V, Liu C, Louder MK, Shi W, Leung K, Yang ES, West A, Gully KL, Stevens LJ, Wang N, Wrapp D, Doria-Rose NA, Stewart-Jones G, Bennett H, et al. 2020. SARS-CoV-2 mRNA vaccine design enabled by prototype pathogen preparedness. Nature 586:567–571. doi:10.1038/s41586-020-2622-0. 32756549 PMC7581537

[21] Polack FP, Thomas SJ, Kitchin N, Absalon J, Gurtman A, Lockhart S, Perez JL, Pérez Marc G, Moreira ED, Zerbini C, Bailey R, Swanson KA, Roychoudhury S, Koury K, Li P, Kalina WV, Cooper D, Frenck RW, Hammitt LL, Türeci Ö, Nell H, Schaefer A, Ünal S, Tresnan DB, Mather S, Dormitzer PR, Şahin U, Jansen KU, Gruber WC, C4591001 Clinical Trial Group. 2020. Safety and efficacy of the BNT162b2 mRNA Covid-19 vaccine. N Engl J Med 383:2603–2615. doi:10.1056/NEJMoa2034577. 33301246 PMC7745181

[22] Barrett ADT. 2017. Yellow fever live attenuated vaccine: a very successful live attenuated vaccine but still we have problems controlling the disease. Vaccine 35:5951–5955. doi:10.1016/j.vaccine.2017.03.032. 28366605

[23] Stokes A, Bauer JH, Hudson NP, Mortimer PP. 2001. The transmission of yellow fever to Macacus rhesus. Rev Med Virol 11:141–148. doi:10.1002/rmv.311. 11376477

[24] Pulendran B. 2009. Learning immunology from the yellow fever vaccine: innate immunity to systems vaccinology. Nat Rev Immunol 9:741–747. doi:10.1038/nri2629. 19763148

[25] Johnson BA, Xie X, Bailey AL, Kalveram B, Lokugamage KG, Muruato A, Zou J, Zhang X, Juelich T, Smith JK, Zhang L, Bopp N, Schindewolf C, Vu M, Vanderheiden A, Winkler ES, Swetnam D, Plante JA, Aguilar P, Plante KS, Popov V, Lee B, Weaver SC, Suthar MS, Routh AL, Ren P, Ku Z, An Z, Debbink K, Diamond MS, Shi P-Y, Freiberg AN, Menachery VD. 2021. Loss of furin cleavage site attenuates SARS-CoV-2 pathogenesis. Nature 591:293–299. doi:10.1038/s41586-021-03237-4. 33494095 PMC8175039

[26] Wang Y, Yang C, Song Y, Coleman JR, Stawowczyk M, Tafrova J, Tasker S, Boltz D, Baker R, Garcia L, Seale O, Kushnir A, Wimmer E, Mueller S. 2021. Scalable live-attenuated SARS-CoV-2 vaccine candidate demonstrates preclinical safety and efficacy. Proc Natl Acad Sci USA 118:e2102775118. doi:10.1073/pnas.2102775118. 34193524 PMC8307828

[27] Trimpert J, Dietert K, Firsching TC, Ebert N, Thi Nhu Thao T, Vladimirova D, Kaufer S, Labroussaa F, Abdelgawad A, Conradie A, Höfler T, Adler JM, Bertzbach LD, Jores J, Gruber AD, Thiel V, Osterrieder N, Kunec D. 2021. Development of safe and highly protective live-attenuated SARS-CoV-2 vaccine candidates by genome recoding. Cell Rep 36:109493. doi:10.1016/j.celrep.2021.109493. 34320400 PMC8289629

[28] Nouailles G, Adler JM, Pennitz P, Peidli S, Teixeira Alves LG, Baumgardt M, Bushe J, Voss A, Langenhagen A, Langner C, Martin Vidal R, Pott F, Kazmierski J, Ebenig A, Lange MV, Mühlebach MD, Goekeri C, Simmons S, Xing N, Abdelgawad A, Herwig S, Cichon G, Niemeyer D, Drosten C, Goffinet C, Landthaler M, Blüthgen N, Wu H, Witzenrath M, Gruber AD, Praktiknjo SD, Osterrieder N, Wyler E, Kunec D, Trimpert J. 2023. Live-attenuated vaccine sCPD9 elicits superior mucosal and systemic immunity to SARS-CoV-2 variants in hamsters. Nat Microbiol doi:10.1038/s41564-023-01352-8. PMC10159847 37012419

[29] Silvas JA, Vasquez DM, Park J-G, Chiem K, Allué-Guardia A, Garcia-Vilanova A, Platt RN, Miorin L, Kehrer T, Cupic A, Gonzalez-Reiche AS, van Bakel H, García-Sastre A, Anderson T, Torrelles JB, Ye C, Martinez-Sobrido L. 2021. Contribution of SARS-CoV-2 accessory proteins to viral pathogenicity in K18 human ACE2 transgenic mice. J Virol 95:e00402-21. doi:10.1128/JVI.00402-21. 34133899 PMC8354228

[30] Ye C, Chiem K, Park J-G, Oladunni F, Platt RN, Anderson T, Almazan F, de la Torre JC, Martinez-Sobrido L. 2020. Rescue of SARS-CoV-2 from a single bacterial artificial chromosome. mBio 11:e0216820. doi:10.1128/mBio.02168-20. PMC7520601 32978313

[31] Oladunni FS, Park J-G, Pino PA, Gonzalez O, Akhter A, Allué-Guardia A, Olmo-Fontánez A, Gautam S, Garcia-Vilanova A, Ye C, Chiem K, Headley C, Dwivedi V, Parodi LM, Alfson KJ, Staples HM, Schami A, Garcia JI, Whigham A, Platt RN, Gazi M, Martinez J, Chuba C, Earley S, Rodriguez OH, Mdaki SD, Kavelish KN, Escalona R, Hallam CRA, Christie C, Patterson JL, Anderson TJC, Carrion R, Dick EJ, Hall-Ursone S, Schlesinger LS, Alvarez X, Kaushal D, Giavedoni LD, Turner J, Martinez-Sobrido L, Torrelles JB. 2020. Lethality of SARS-CoV-2 infection in K18 human angiotensin-converting enzyme 2 transgenic mice. Nat Commun 11:6122. doi:10.1038/s41467-020-19891-7. 33257679 PMC7705712

[32] Hassan AO, Case JB, Winkler ES, Thackray LB, Kafai NM, Bailey AL, McCune BT, Fox JM, Chen RE, Alsoussi WB, Turner JS, Schmitz AJ, Lei T, Shrihari S, Keeler SP, Fremont DH, Greco S, McCray PB, Perlman S, Holtzman MJ, Ellebedy AH, Diamond MS. 2020. A SARS-CoV-2 infection model in mice demonstrates protection by neutralizing antibodies. Cell 182:744–753.e744. doi:10.1016/j.cell.2020.06.011. 32553273 PMC7284254

[33] Ye CJ, Chiem K, Park JG, Silvas JA, Morales Vasquez D, Sourimant J, Lin MJ, Greninger AL, Plemper RK, Torrelles JB, Kobie JJ, Walter MR, de la Torre JC, Martinez-Sobrido L. 2021. Analysis of SARS-CoV-2 infection dynamic in vivo using reporter-expressing viruses. Proc Natl Acad Sci USA 118:e2111593118. doi:10.1073/pnas.2111593118. 34561300 PMC8521683

[34] Sia SF, Yan L-M, Chin AWH, Fung K, Choy K-T, Wong AYL, Kaewpreedee P, Perera RAPM, Poon LLM, Nicholls JM, Peiris M, Yen H-L. 2020. Pathogenesis and transmission of SARS-CoV-2 in golden hamsters. Nature 583:834–838. doi:10.1038/s41586-020-2342-5. 32408338 PMC7394720

[35] Monto AS. 2021. The future of SARS-CoV-2 vaccination—lessons from influenza. N Engl J Med 385:1825–1827. doi:10.1056/NEJMp2113403. 34739199

[36] McCray PB, Pewe L, Wohlford-Lenane C, Hickey M, Manzel L, Shi L, Netland J, Jia HP, Halabi C, Sigmund CD, Meyerholz DK, Kirby P, Look DC, Perlman S, Jr. 2007. Lethal infection of K18-hACE2 mice infected with severe acute respiratory syndrome coronavirus. J Virol 81:813–821. doi:10.1128/JVI.02012-06. 17079315 PMC1797474

[37] Winkler ES, Bailey AL, Kafai NM, Nair S, McCune BT, Yu J, Fox JM, Chen RE, Earnest JT, Keeler SP, Ritter JH, Kang L-I, Dort S, Robichaud A, Head R, Holtzman MJ, Diamond MS. 2020. SARS-CoV-2 infection of human ACE2-transgenic mice causes severe lung inflammation and impaired function. Nat Immunol 21:1327–1335. doi:10.1038/s41590-020-0778-2. 32839612 PMC7578095

[38] Israelow B, Mao T, Klein J, Song E, Menasche B, Omer SB, Iwasaki A. 2021. Adaptive immune determinants of viral clearance and protection in mouse models of SARS-CoV-2. Sci Immunol 6:eabl4509. doi:10.1126/sciimmunol.abl4509. 34623900 PMC9047536

[39] Zhu N, Zhang D, Wang W, Li X, Yang B, Song J, Zhao X, Huang B, Shi W, Lu R, Niu P, Zhan F, Ma X, Wang D, Xu W, Wu G, Gao GF, Tan W, China Novel Coronavirus Investigating and Research Team. 2020. A novel coronavirus from patients with pneumonia in China, 2019. N Engl J Med 382:727–733. doi:10.1056/NEJMoa2001017. 31978945 PMC7092803

[40] Chiem K, Ye C, Martinez-Sobrido L. 2020. Generation of recombinant SARS-CoV-2 using a bacterial artificial chromosome. Curr Protoc Microbiol 59:e126. doi:10.1002/cpmc.126. 33048448 PMC7646048

[41] Bolger AM, Lohse M, Usadel B. 2014. Trimmomatic: a flexible trimmer for Illumina sequence data. Bioinformatics 30:2114–2120. doi:10.1093/bioinformatics/btu170. 24695404 PMC4103590

[42] Langmead B, Salzberg SL. 2012. Fast gapped-read alignment with Bowtie 2. Nat Methods 9:357–359. doi:10.1038/nmeth.1923. 22388286 PMC3322381

[43] Pedersen BS, Quinlan AR. 2018. Mosdepth: quick coverage calculation for genomes and exomes. Bioinformatics 34:867–868. doi:10.1093/bioinformatics/btx699. 29096012 PMC6030888

[44] Wilm A, Aw PPK, Bertrand D, Yeo GHT, Ong SH, Wong CH, Khor CC, Petric R, Hibberd ML, Nagarajan N. 2012. LoFreq: a sequence-quality aware, ultra-sensitive variant caller for uncovering cell-population heterogeneity from high-throughput sequencing datasets. Nucleic Acids Res 40:11189–11201. doi:10.1093/nar/gks918. 23066108 PMC3526318

[45] Cingolani P, Platts A, Wang LL, Coon M, Nguyen T, Wang L, Land SJ, Lu X, Ruden DM. 2012. A program for annotating and predicting the effects of single nucleotide polymorphisms, SnpEff: SNPs in the genome of Drosophila melanogaster strain w(1118); iso-2; iso-3. Fly (Austin) 6:80–92. doi:10.4161/fly.19695. 22728672 PMC3679285

[46] Chiem K, Morales Vasquez D, Silvas JA, Park J-G, Piepenbrink MS, Sourimant J, Lin MJ, Greninger AL, Plemper RK, Torrelles JB, Walter MR, de la Torre JC, Kobie JK, Ye C, Martinez-Sobrido L. 2021. A bifluorescent-based assay for the identification of neutralizing antibodies against SARS-CoV-2 variants of concern in vitro and in vivo. J Virol 95:e0112621. doi:10.1128/JVI.01126-21. 34495697 PMC8549516

[47] Park J-G, Oladunni FS, Chiem K, Ye C, Pipenbrink M, Moran T, Walter MR, Kobie J, Martinez-Sobrido L. 2021. Rapid in vitro assays for screening neutralizing antibodies and antivirals against SARS-CoV-2. J Virol Methods 287:113995. doi:10.1016/j.jviromet.2020.113995. 33068703 PMC7554492

[48] Jensen EC. 2013. Quantitative analysis of histological staining and fluorescence using ImageJ. Anat Rec (Hoboken) 296:378–381. doi:10.1002/ar.22641. 23382140

