# Supplementary material for: Immunization with Recombinant Accessory Protein-Deficient SARS-CoV-2 Protects against Lethal Challenge and Viral Transmission
Source: Microbiol Spectr. 2023 May 16;11(3):e00653-23. doi: 10.1128/spectrum.00653-23 (PMC10269623; doi:10.1128/spectrum.00653-23)
Supplement: Supplemental file 5 — Legends to Fig. S1 to S4. Download spectrum.00653-23-s0005.pdf, PDF file, 0.4 MB [file spectrum.00653-23-s0005.pdf]

Figure S1. Induction of cytokines and chemokines in lung homogenates, and activation of cytokine positive CD4 and CD8 splenocytes against viral components upon double ORF-deficient rSARS-CoV-2 infection.

(A) Cytokine and chemokine levels were measured in triplicate in the lung homogenates of K18 hACE2 transgenic mice infected ($2x10^5$ PFU/mouse) with WT or double ORF-deficient rSARS-CoV-2 at 2 and 4 dpi. Data are presented as mean ± SEM, and comparisons of the means between indicated groups are analyzed by One-way ANOVA. *, $P<0.05$; and **, $P<0.01$.

(B) Intracellular cytokine positive $CD4^+$ T cells in the splenocytes of the double ORF-deficient rSARS-CoV-2-infected K18 hACE2 transgenic mice were analyzed after stimulation of S1 peptide pool, E, and M using flow cytometry. The splenocytes from the two surviving K18 hACE2 transgenic mice infected with rSARS-CoV-2 WT (n=5) for 21 days were collected as a positive control. Data are presented as mean ± SEM.

(C) Intracellular cytokine positive $CD8^+$ T cells in the splenocytes of the double ORF-deficient rSARS-CoV-2-infected K18 hACE2 transgenic mice were analyzed after stimulation of S1 peptide pool, E, and M using flow cytometry. The splenocytes from the two surviving K18 hACE2 transgenic mice infected with rSARS-CoV-2 WT (n=5) for 21 days were collected as a positive control. Data are presented as mean ± SEM.

Figure S2. Analysis of mCherry expression, pathological lesions, viral replications, and cytokine and chemokines induction in the lungs of mock- or rSARS-CoV-2 Δ3a/Δ7b-vaccinated K18 hACE2 transgenic mice at 2 and 4 days post-challenge with rSARS-CoV-2 mCherryNluc.

(A) Expression of mCherry in the lungs of K18 hACE2 transgenic mice challenged with rSARS-CoV-2 mCherryNluc.

(B) Pathological lesions on the lungs surface of K18 hACE2 transgenic mice challenged with rSARS-CoV-2 mCherryNluc.

(C) Quantitative analysis of mCherry intensity in the lungs of K18 hACE2 transgenic mice challenged with rSARS-CoV-2 mCherryNluc by Aura program. Data are presented as mean ± SD, and comparisons of the means between indicated groups are analyzed by One-way ANOVA. *, P<0.05; and ns, not significant.

(D) Quantitative analysis of gross pathological lesion on the lungs surface of challenged K18 hACE2 transgenic mice by Image J. Data are presented as mean ± SD, and comparisons of the means between indicated groups are analyzed by One-way ANOVA. *, P<0.05; **, P<0.01; and ns, not significant.

(E) Cytokine and chemokine levels were measured (triplicate) in the lung homogenates of K18 hACE2 transgenic mice at 2 and 4 days post-challenge with rSARS-CoV-2 mCherryNluc. Data are presented as mean ± SEM, and comparisons of the means between indicated groups are analyzed by ANOVA. *, P<0.05; **, P<0.01; and ns, not significant.

Figure S3. Analysis of the mCherry expression, viral replication, and Nluc activity in the lungs of the double ORF-deficient rSARS-CoV-2-vaccinated hamsters challenged and co-housed with susceptible contact hamsters.

(A) Expression of mCherry in the lungs of challenged and contact hamsters.

(B) Quantitative analysis of mCherry intensity in the lungs of challenged and contact hamsters by Aura program. Data are presented as mean ± SD, and comparisons of the means between indicated groups are analyzed by One-way ANOVA. **, *P*<0.01.

Figure S4. Analysis of the mCherry expression, virus replication, and Nluc activity in the lungs of rSARS-CoV-2 mCherryNluc-infected donor hamsters and their contacts.

(A) Expression of mCherry in the lungs of infected donor and vaccinated contact hamsters.

(B) Quantitative analysis of mCherry intensity in the lungs of infected donor and vaccinated contact hamsters Aura program. Data are presented as mean ± SD, and comparisons of the means between indicated groups are analyzed by One-way ANOVA. **, *P*<0.01.
